# Branched-chain amino acid metabolism, insulin sensitivity and liver fat response to exercise training in sedentary dysglycaemic and normoglycaemic men

**DOI:** 10.1007/s00125-020-05296-0

**Published:** 2020-10-29

**Authors:** Sindre Lee, Hanne L. Gulseth, Torgrim M. Langleite, Frode Norheim, Thomas Olsen, Helga Refsum, Jørgen Jensen, Kåre I. Birkeland, Christian A. Drevon

**Affiliations:** 1 Department of Nutrition, Institute of Basic Medical Sciences, Faculty of Medicine, University of Oslo, Oslo, Norway; 2 Institute of Clinical Medicine, Faculty of Medicine, University of Oslo, Oslo, Norway; 3 Department of Transplantation Medicine, Oslo University Hospital, Oslo, Norway; 4 Department of Chronic Diseases and Ageing, Norwegian Institute of Public Health, Oslo, Norway; 5 Norwegian School of Sport Sciences, Oslo, Norway

**Keywords:** Adipose tissue, Branched-chain amino acids, Catabolism, Diabetes, Exercise, Insulin resistance, Insulin sensitivity, Muscle

## Abstract

**Aims/hypothesis:**

Obesity and insulin resistance may be associated with elevated plasma concentration of branched-chain amino acids (BCAAs) and impaired BCAA metabolism. However, it is unknown whether the insulin-sensitising effect of long-term exercise can be explained by concomitant change in BCAAs and their metabolism.

**Methods:**

We included 26 sedentary overweight and normal-weight middle-aged men from the MyoGlu clinical trial, with or without dysglycaemia, for 12 weeks of supervised intensive exercise intervention, including two endurance and two resistance sessions weekly. Insulin sensitivity was measured as the glucose infusion rate (GIR) from a hyperinsulinaemic−euglycaemic clamp. In addition, maximum oxygen uptake, upper and lower body strength and adipose tissue depots (using MRI and spectroscopy) were measured, and subcutaneous white adipose tissue (ScWAT) and skeletal muscle (SkM) biopsies were harvested both before and after the 12 week intervention. In the present study we have measured plasma BCAAs and related metabolites using CG-MS/MS and HPLC-MS/MS, and performed global mRNA-sequencing pathway analysis on ScWAT and SkM.

**Results:**

In MyoGlu, men with dysglycaemia displayed lower GIR, more fat mass and higher liver fat content than normoglycaemic men at baseline, and 12 weeks of exercise increased GIR, improved body composition and reduced liver fat content similarly for both groups. In our current study we observed higher plasma concentrations of BCAAs (14.4%, *p* = 0.01) and related metabolites, such as 3-hydroxyisobutyrate (19.4%, *p* = 0.034) in dysglycaemic vs normoglycaemic men at baseline. Baseline plasma BCAA levels correlated negatively to the change in GIR (ρ = −0.41, *p* = 0.037) and $\dot{V}{\mathrm{O}}_{2\max }$ (ρ = −0.47, *p* = 0.015) after 12 weeks of exercise and positively to amounts of intraperitoneal fat (ρ = 0.40, *p* = 0.044) and liver fat (ρ = 0.58, *p* = 0.01). However, circulating BCAAs and related metabolites did not respond to 12 weeks of exercise, with the exception of isoleucine, which increased in normoglycaemic men (10 μmol/l, *p* = 0.01). Pathway analyses of mRNA-sequencing data implied reduced BCAA catabolism in both SkM and ScWAT in men with dysglycaemia compared with men with normoglycaemia at baseline. Gene expression levels related to BCAA metabolism correlated positively with GIR and markers of mitochondrial content in both SkM and ScWAT, and negatively with fat mass generally, and particularly with intraperitoneal fat mass. mRNA-sequencing pathway analysis also implied increased BCAA metabolism after 12 weeks of exercise in both groups and in both tissues, including enhanced expression of the gene encoding branched-chain α-ketoacid dehydrogenase (BCKDH) and reduced expression of the BCKDH phosphatase in both groups and tissues. Gene expression of *SLC25A44*, which encodes a mitochondrial BCAA transporter, was increased in SkM in both groups, and gene expression of *BCKDK*, which encodes BCKDH kinase, was reduced in ScWAT in dysglycaemic men. Mediation analyses indicated a pronounced effect of enhanced SkM (~53%, *p* = 0.022), and a moderate effect of enhanced ScWAT (~18%, *p* = 0.018) BCAA metabolism on improved insulin sensitivity after 12 weeks of exercise, based on mRNA sequencing. In comparison, plasma concentration of BCAAs did not mediate any effect in this regard.

**Conclusion/interpretation:**

Plasma BCAA concentration was largely unresponsive to long-term exercise and unrelated to exercise-induced insulin sensitivity. On the other hand, the insulin-sensitising effect of long-term exercise in men may be explained by enhanced SkM and, to a lesser degree, also by enhanced ScWAT BCAA catabolism.

**Figure:**
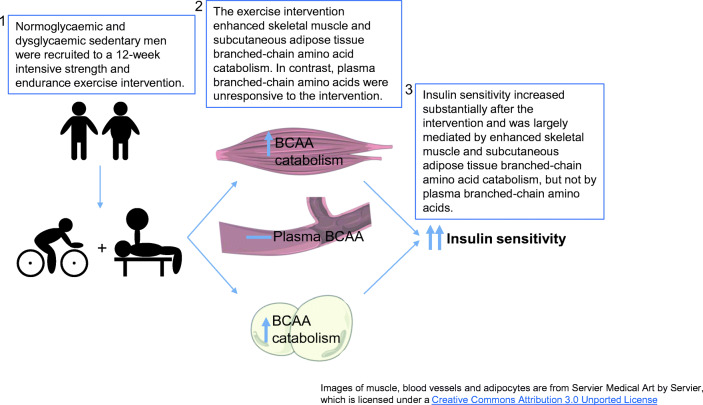
Graphical abstract

**Electronic supplementary material:**

The online version of this article (10.1007/s00125-020-05296-0) contains peer-reviewed but unedited supplementary material, which is available to authorised users.

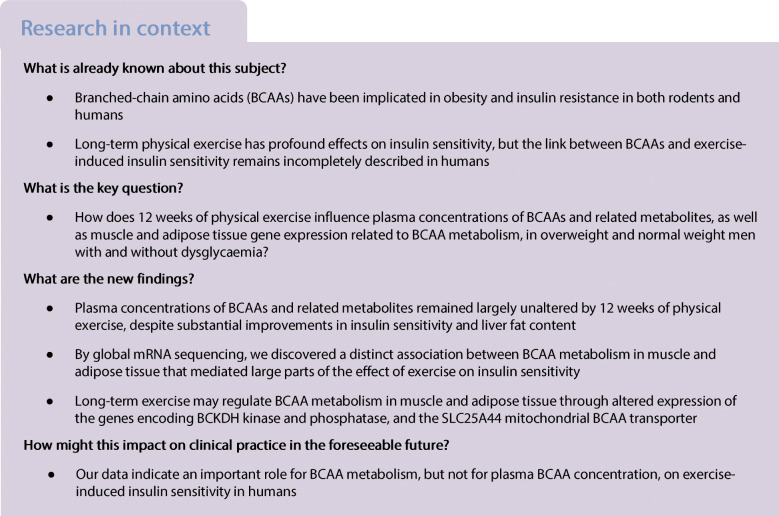



## Introduction

Accumulation of the branched-chain amino acids (BCAAs) valine, leucine and isoleucine in plasma were already linked to obesity and insulin resistance in the 1960s [1]. More recently, large prospective epidemiological studies applying plasma metabolomic profiling have shown that a plasma BCAA and aromatic amino acid signature at baseline could predict future type 2 diabetes and insulin resistance [1–9].

Accumulation of plasma BCAAs may be caused by several processes influencing their rate of appearance and disappearance in the circulation [10]. Amino acids from diet or tissues may enhance plasma BCAA concentration, whereas increased uptake and oxidation, such as after acute exercise, may decrease plasma BCAA concentration [10]. Increased plasma concentration of BCAAs may reflect suppressed BCAA catabolism in peripheral tissues [11–13]. Mouse studies have suggested that suppressed BCAA degradation can induce insulin resistance and that restoring flux through the BCAA degradation pathway may increase insulin sensitivity [14]. Furthermore, increased catabolism of BCAAs in the gut microbiome of responders to 12 weeks of combined endurance and strength exercise led to lower plasma concentrations of BCAAs and improved insulin sensitivity [15]. However, another study on nine overweight participants undergoing 6 months of combined endurance and strength exercise improved insulin sensitivity with no alteration of circulating BCAAs [16].

The large amino acid transporter (LAT1) mediates tissue uptake of BCAAs, tryptophan, tyrosine and phenylalanine [17, 18]. Inside cells, BCAAs may be degraded by a complex consisting of 43 enzymes located mainly in mitochondria [19], which plays a pivotal role in BCAA homeostasis in humans [11]. The flux-generating step in BCAA degradation is the branched-chain α-ketoacid dehydrogenase (BCKDH) [19]*.* The highest BCKDH activity/BCAA degradation capacity is found in adipose tissue, liver and skeletal muscle (SkM) [19–21]. After oxidation by BCKDH, BCAAs are trapped within mitochondria, with the exception of 3-hydroxyisobutyrate (3-HIB), which may serve as a marker of valine degradation [19]. Resulting carbons from catabolised BCAAs depend on the tricarboxylic acid (TCA) cycle for complete oxidation [19].

As an additional analysis from the MyoGlu trial (ClinicalTrials.gov registration no. NCT01803568) [22, 23], we have now investigated whether the insulin-sensitising effect of exercise could be explained by plasma BCAAs and their metabolism in SkM and subcutaneous white adipose tissue (ScWAT) among overweight and normal-weight sedentary men, with or without dysglycaemia. A relationship between BCAAs and aromatic amino acids has been suggested in the pathology of insulin resistance due to their shared reliance on LAT1 [17, 18]. A secondary aim was to investigate aromatic amino acids in the context of exercise and insulin sensitivity.

## Methods

### The MyoGlu clinical trial

**Fig. 1:**
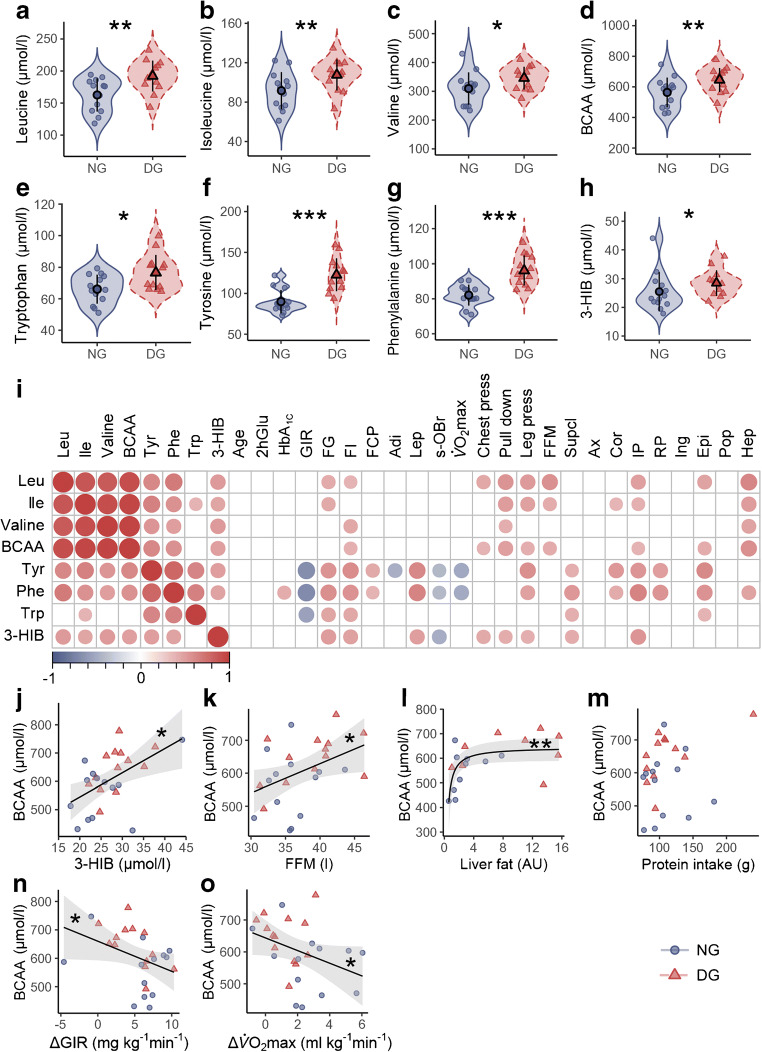
BCAAs and aromatic amino acids at baseline. (**a**–**c**) Concentrations of individual BCAAs, and (**d**) their molar sum; (**e**–**g**) concentrations of aromatic amino acids and (**h**) 3-HIB in NG vs DG men. The violin plots depict means ± SD together with the data distribution (shaded areas). (**i**) A Spearman’s correlation heat map between plasma amino acids, 3-HIB and relevant phenotypes. The size of the dot indicates the degree of correlation. The colour key below the heat map indicates positive (red) and negative (blue) correlations. Non-significant correlations are not shown. (**j**–**m**) Linear regressions between the molar sum of baseline plasma BCAAs and factors potentially influencing their concentration. (**n**–**o**) Regression analyses between plasma BCAA levels at baseline and subsequent response in insulin sensitivity and $\dot{V}{\mathrm{O}}_{2\max }$ to 12 weeks of exercise. The shaded areas indicate 95% CI of the correlation trend lines. *p < 0.05, **p < 0.01 and ***p < 0.001. 2hGlu, 2 h glucose after an OGTT; F, fasting; G, glucose; I, insulin; CP, C-peptide; Adi, high-molecular mass adiponectin; Lep, leptin; s-OBr, soluble leptin receptor; Supcl, supraclavicular fat; Ax, axillary fat; Cor, cordial fat; IP, intraperitoneal fat; RP, retroperitoneal fat; Ing, inguinal fat; Epi, epididymal fat; Pop, popliteal fat; Hep, hepatic fat; AU, arbitrary units; chest press, pull down and leg press refer to the maximum weight that the participants can manage for each exercise

MyoGlu was a controlled clinical trial (clinicaltrials.gov: NCT01803568) and has been described in detail previously [22, 23]. MyoGlu adhered to the Declaration of Helsinki and the National Regional Committee for Medical and Health Research Ethics North, Tromsø, Norway approved the study, with reference number: 2011/882. We obtained written informed consent from all participants before any study-related procedure. A study flow chart and the study design are presented in electronic supplementary material (ESM) Fig. 1.

The study participants included 26 sedentary (<1 exercise session/week) men aged 40 to 65 years of Scandinavian origin, who were recruited to the dysglycaemia (DG) group (*n* = 13; fasting glucose ≥5.6 mmol/l and/or 2 h glucose ≥7.8 mmol/l and/or insulin resistance [HOMA-IR >2.0]; with BMI 26.8 to 32.5 kg/m^2^) and normoglycaemia (NG) group (*n* = 13; fasting glucose <5.6 mmol/l and 2 h glucose <7.8 mmol/l; with BMI 20.9 to 26.7 kg/m^2^). Exclusion criteria included family history of diabetes (for NG men only), hypertension, liver or kidney disease, chronic inflammatory diseases or any medication known to affect glucose metabolism.

The participants were instructed not to change their diet and registered their habitual diet in an extensively validated food frequency questionnaire (FFQ) [24, 25]. FFQs were analysed using the food database AE-10 and the Norwegian KBS food and nutrients calculation system KBS v.7.1 (Kostberegningssystem, Oslo, Norway). Up to two units of alcohol intake per day was allowed, and water was consumed freely. The participants consumed a standardised meal after overnight fasting 90–120 min before the bicycle challenges. The meal was carbohydrate-rich including bread, apple juice, cheese and jam, providing ~23% of estimated total daily energy expenditure.

In the long-term exercise arm of MyoGlu the participants performed 4 h of intensive exercise weekly for 12 weeks under professional supervision (two whole-body strength training sessions and two bicycle interval sessions [using cycle ergometers] each session lasting 1 h). The 12 week intervention included linear progression in workload for both strength and endurance exercises [22, 23]. The acute exercise arm of MyoGlu included a 45 min bicycle challenge (also using cycle ergometers) at 70% $\dot{V}{\mathrm{O}}_{2\max }$ performed both before and after the 12 week intervention.

$\dot{V}{\mathrm{O}}_{2\max }$ was tested both before and after the 12 week intervention for each participant by cycling for 1 min followed by 15 W increase in workload every 30 s until exhaustion [22, 23]. A successful test was based on O_2_ consumption increasing <0.5 ml kg^−1^ min^−1^ over a 30 W increase in workload, respiratory exchange ratio >1.10, and blood lactate >7.0 mmol/l.

The hyperinsulinaemic−euglycaemic clamp was performed after overnight fasting with an insulin infusion rate of 40 mU m^–2^ min^–1^ using human insulin (Actrapid, NovoNordisk, Bagsvaerd, Denmark). Infusion of glucose 200 mg/ml was continually adjusted to maintain euglycaemia at 5.0 mmol/l. The clamp was maintained for a minimum of 150 min [26], until at least 30 min of stable euglycaemia was obtained. CVs for measurement of glucose and insulin were 4% and 5%, respectively. Post intervention, each participant had a standardised endurance session 3 days prior to and refrained from physical exercise and alcohol 48 h before the clamp re-tests. Insulin sensitivity was reported as steady state glucose infusion rate (GIR) relative to body weight and relative to mean plasma insulin during the last 30 min of the clamp. Whole blood glucose concentration was measured using glucose oxidase (YSI 2300, Yellow Springs, OH, USA), and plasma glucose concentration was calculated as whole blood glucose × 1.119 [26].

SkM and ScWAT were obtained from *vastus lateralis* muscle and periumbilical subcutaneous adipose tissue, as described previously [22, 23]. After sterilisation, a lidocaine-based local anaesthetic was injected into the skin and subcutis prior to both SkM and ScWAT biopsies. Biopsies were dissected on a cold aluminium plate to remove blood and other materials before freezing. SkM was obtained before, just after and 2 h after the 45 min bicycle challenge both before and after the 12 week intervention. ScWAT was obtained just after the bicycle challenge both before and after the 12 week intervention (ESM Fig. 1).

Body composition was analysed using MRI/MRS performed on a 1.5 T Philips Achieva MR (Best, Netherlands) to measure fat-free mass (FFM) and adipose tissue depots [23]. The ankle-to-neck MRI protocol included a 3D DIXON acquisition providing water and lipid quantification; data were then analysed using the nordicICE software package v.4.0.0 (NordicNeuroLab, Bergen, Norway). MRS monitoring liver fat content was performed using a single voxel spectroscopy acquisition. A 15 × 10× 25 mm^3^ voxel was placed in a homogeneous area taking care to avoid any visible fat or fascia. Scan parameters were TR/TE: 3000/31.2 ms bandwidth: 2500 Hz, no. of samples: 4096, no. of acquisitions: 64. Data were analysed using the jMRUI v.5.2 workflow (www.jmrui.eu).

### mRNA-sequencing analyses

Frozen biopsies were crushed to powder by a pestle and a mortar cooled with liquid nitrogen. Frozen biopsies were transferred into 1 ml QIAzol Lysis Reagent (Qiagen, Hilden, Germany), and homogenised using TissueRuptor (Qiagen) at full speed for 15 s, twice. Total RNA was isolated from the homogenates using miRNeasy Mini Kit (Qiagen). RNA integrity and concentration were determined using Agilent RNA 6000 Nano Chips on a Bioanalyzer 2100 (Agilent Technologies, Santa Clara, CA, USA). RNA was converted to cDNA using a High-Capacity cDNA Reverse Transcription Kit (Applied Biosystems, Foster, CA, USA). The cDNA reaction mixture was diluted in water and cDNA equivalent of 25 ng RNA used for each sample. Sequencing was performed using the Illumina HiSeq 2000 system (San Diego, CA, USA) with multiplex at the Norwegian Sequencing Centre, University of Oslo. Illumina HiSeq real-time analysis v1.17.21.3 was used. Reads passing Illumina’s recommended parameters were demultiplexed using CASAVA v1.8.2 (Illumina). For pre-alignment quality checks, we used the software FastQC v0.10.1 (Illumina). Read-alignment was performed using Tophat v2.0.8 (www.ccb.jhu.edu/software/tophat/index.shtml). Reads were counted by HTSeq v0.6.1 (www.htseq.readthedocs.io/en/master). Validation of our mRNA-sequencing procedure was performed by comparing the results to TaqMan real-time RT-PCR, as described previously [22]. mRNA-sequencing pathway analysis was performed using the native generally applicable gene set enrichments (GAGE) workflow (Kyoto Encyclopaedia of Genes and Genomes [KEGG]) [27]. Parts of the mRNA-sequencing data have been described in conjunction with previous reports on adipokines [22] and myokines [28], but the data included in this paper have not been reported previously.

### Electron microscopy

Selected bundles of muscle biopsies taken just after acute exercise before as well as after 12 weeks of exercise (*n* = 18) were used for electron microscopy analyses, as described in detail previously [29]. Briefly, SkM was embedded in Durcupan, ultrathin sections of 60 nm were cut using an ultramicrotome from Leica (Vienna, Austria) and images were obtained using a Tecnai G2 electron microscope from FEI (Hillsboro, OR, USA). In the current study we counted mitochondria by point counting [30] performed at 6000-fold magnification on five randomly selected areas, total of 400 μm^2^, from each participant using a 150 × 150 lattice. The hit points of mitochondria were marked manually on blinded images and counted. The percentage was estimated by the ratio of hit points/total points.

### Plasma BCAAs and related metabolites

In the current study, we analysed frozen plasma samples from the MyoGlu participants and measured amino acids using HPLC-MS/MS [31], the method has been described previously in regards to sulphur-containing amino acids and exercise [32], and 3-HIB with the use of GC-MS/MS at Bevital (www.bevital.no) [33].

### Statistics

Data analysis was performed using linear mixed model regression with the *lme4* [34] package in R. For variables measured at all time points we constructed a 3 × 2 × 2 factorial design with repeated measures analysing a variable of interest as a function of acute exercise (before, just after and 2 h after) by long-term exercise (before and after 12 weeks) by group (DG and NG) with a random intercept for participants to account for correlated measures. For variables we did not measure during acute exercise, but only in response to 12 weeks of exercise, a 2 × 2 (no data and, thus, no factor for acute exercise) factorial design was constructed in a similar fashion as the aforementioned model.

Spearman’s rank correlation tests were performed to make bivariate correlation heatmaps and linear regression was used to evaluate specific correlations of interest.

Mediation analyses were performed using the R package *mediation* with 1000 permutations and *set.seed(1)* to ensure reproducibility. The null model was the mixed model for GIR, as described above. Next, a model was constructed substituting GIR with the mediator of interest. Finally, a model was constructed similarly to the null model, but including the mediator of interest as a covariate. If both the outcome and the mediator respond to intervention, the mediator might explain the effect of e.g. exercise on GIR. This is then tested in the final model: (1) if the effect of exercise on GIR is reduced and becomes insignificant, the mediator may fully explain exercise-induced GIR; (2) if the effect of exercise on GIR is reduced, but remains significant, the mediator is a partial mediator of exercise-induced GIR; (3) if the effect of exercise on GIR remains the same despite the presence of the potential mediator, then this variable is not a mediator.

Normal distribution of residuals was assessed visually and, if necessary, a natural log-transformation was applied to approximate normality. *p* values were considered significant at α = 0.05. Correction for multiple testing was performed using a control of the false discovery rate [35], set at 10%. All data were analysed using R v.3.6.0 [36].

## Results

### Insulin sensitivity and responses to 12 weeks of exercise

Participant characteristics are presented in Table 1, as reported previously [22, 23]. Briefly, GIR was ~45% lower in DG vs NG men at baseline and increased in response to 12 weeks of exercise by ~45% and ~38% in DG and NG men, respectively. DG men had significantly more adipose tissue and hepatic fat, hyperinsulinaemia and higher HbA_1c_ levels compared with NG men. Body composition and physical fitness improved significantly for both groups in response to 12 weeks of exercise. We observed no differences in dietary factors between groups or before vs after 12 weeks of exercise (ESM Table 1).

**Table 1:** Participant characteristics and responses to 12 weeks of exercise intervention

Characteristic	Baseline				12 weeks				Change			
	NG	n	DG	n	NG	n	DG	n	ΔNG	n	ΔDG	n
Age (years)	50 (7.4)	13	53 (5.6)	13	NA	NA	NA	NA	NA	NA	NA	NA
HbA_1c_ (%)	5.2 (0.4)	13	5.5 (0.4)*	13	NA	NA	NA	NA	NA	NA	NA	NA
HbA_1c_ (mmol/mol)	33 (4.4)	13	37 (4.3)*	13	NA	NA	NA	NA	NA	NA	NA	NA
2 h glucose (mmol/l)	5.0 (1.3)	13	6.7 (2.8)	13	NA	NA	NA	NA	NA	NA	NA	NA
Weight (kg)	79 (8.2)	13	95 (10)***	13	78 (8.2)	13	94 (9.7)***	13	−0.27 (1.6)	13	−1.7 (2.2)†	13
BMI (kg/m^2^)	24 (2.0)	13	29 (2.4)***	13	24 (1.8)	13	29 (2.3)***	13	−0.023 (0.47)	13	−0.38 (1.2)	13
FFM (l)	36 (3.6)	13	39 (5.1)	13	38 (3.6)	13	41 (5.2)	13	2.3 (1.3)†††	13	2.0 (1.0)†††	13
GIR (mg kg^−1^ min^−1^)	7.6 (1.6)	13	4.2 (1.8)***	13	10 (2.6)	13	5.4 (1.8)***	13	2.7 (2.0)†††	13	1.2 (1.1)††	13
Clamp-insulin (pmol/l)	444 (132)	13	456 (73)	13	451 (103)	13	512 (107)	13	6.6 (85)	13	56 (88)†	13
GIR/I	1.8 (0.64)	13	0.95 (0.45)***	13	2.4 (0.82)	13	1.1 (0.40)***	13	0.59 (0.52)††	13	0.15 (0.23)†	13
F-insulin (pmol/l)	39 (19)	13	65 (27)**	13	39 (12)	13	77 (31)***	13	0.24 (20)	13	12 (28)	13
F-c-peptide (pmol/l)	588 (118)	13	933 (249)***	13	618 (124)	13	977 (197)***	13	30 (154)	13	44 (242)	13
Leptin (ng/ml)	7.9 (1.9)	13	16 (6.5)***	13	7.2 (1.9)	13	13 (6.0)**	13	−0.72 (1.2)†	13	−3.4 (2.6)†††	13
s-OBr (ng/ml)	5.6 (1.5)	13	4.4 (0.67)*	13	5.3 (1.4)	13	4.4 (0.66)*	13	−0.26 (0.78)	13	−0.039 (0.26)	13
$\dot{V}{\mathrm{O}}_{2\max }$(ml kg^−1^ min^−1^)	44 (4.4)	13	37 (4.9)*	13	50 (5.1)	13	42 (5.0)***	13	5.7 (4.1)†††	13	4.8 (2.8)†††	13
Chest press (kg)^a^	66 (17)	13	69 (14)	13	77 (20)	12	77 (13)	13	12 (5.7)†††	12	8.7 (3.9)†††	13
Pull down (kg)^a^	69 (9.3)	13	76 (15)	13	80 (9.6)	12	85 (14)	13	12 (5.6)†††	12	9.6 (4.1)†††	13
Leg press (kg)^a^	200 (37)	13	249 (30)***	13	218 (38)	13	278 (28)***	13	18 (13)†††	13	30 (17)†††	13
Total fat (l)	31 (4.6)	13	45 (7.8)***	13	29 (4.1)	13	42 (6.7)***	13	−2.3 (1.4)†††	13	−3.6 (2.8)†††	13
Supraclavicular fat (AU)	87 (35)	12	130 (35)**	12	82 (26)	12	126 (39)**	11	−4.3 (19)	12	−1.2 (21)	11
Axillary fat (AU)	185 (66)	13	295 (108)**	13	177 (47)	13	261 (83)**	13	−8.2 (34)	13	−34 (53)†	13
Pericardial fat (AU)	113 (48)	13	159 (61)*	13	118 (45)	13	156 (55)	13	5.1 (15)	13	−2.5 (18)	13
Intraperitoneal fat (AU)	961 (645)	13	2417 (718)***	13	730 (537)	13	1830 (819)***	13	−231 (237)††	13	−587 (332)†††	13
Retroperitoneal fat (AU)	1010 (365)	13	1971 (634)***	13	875 (306)	13	1765 (595)***	13	−135 (117)††	13	−207 (161)†††	13
Inguinal fat (AU)	76 (23)	13	112 (31)**	13	74 (26)	13	103 (25)**	13	−1.5 (7.7)	13	−8.9 (24)	13
Epidydimal fat (AU)	5.3 (1.5)	13	12 (6.9)**	13	5.6 (1.7)	13	13 (7.5)**	13	0.31 (1.0)	13	0.31 (2.0)	13
Popliteal fat (AU)	135 (27)	13	199 (84)*	12	135 (35)	13	194 (78)*	12	0.15 (22)	13	−5.1 (33)	12
Pancreatic fat (AU)	0.084 (0.071)	10	0.38 (0.47)	8	0.047 (0.040)	10	0.19 (0.16)*	8	−0.037 (0.054)	10	−0.18 (0.46)	8
Hepatic fat (AU)	2.8 (2.2)	10	9.1 (5.9)*	9	2.2 (2.4)	10	6.5 (4.2)*	9	−0.58 (1.1)	10	−2.7 (2.6)†	9

Data are mean (SD)

Participant characteristics from MyoGlu have been reported previously [22, 23]

^a^Chest press, pull down and leg press values refer to the maximum weight that the participants can manage for each exercise

*p < 0.05, **p < 0.01 and ***p < 0.001 DG vs NG; †*p* < 0.05, ††*p* < 0.01 and †††*p* < 0.001 response to 12 weeks of exercise

AU, arbitrary units; Clamp-insulin, mean plasma insulin during the last 30 min of the clamp; F, fasting; GIR/I, GIR relative to clamp-insulin × 100; NA, not available; s-OBr, soluble leptin receptor

### Effect of dysglycaemia at baseline

Plasma concentrations of leucine (Fig. 1a), isoleucine (Fig. 1b) and valine (Fig. 1c), and their molar sum (14.4%, *p* = 0.01, Fig. 1d), as well as tryptophan (Fig. 1e), tyrosine (Fig. 1f) and phenylalanine (Fig. 1g) were elevated in DG vs NG men at baseline. The valine degradation marker 3-HIB was also elevated in DG vs NG men at baseline (19.4%, *p* = 0.034, Fig. 1h).

All BCAAs, aromatic amino acids (with the exception of tryptophan) and 3-HIB were highly intercorrelated and correlated negatively with several glucometabolic traits and positively to markers of adiposity (Fig. 1i), such as intraperitoneal (ρ = 0.40, *p* = 0.044) and liver fat content (ρ = 0.58, *p* = 0.01). Aromatic amino acids displayed the strongest negative correlations to GIR and $\dot{V}{\mathrm{O}}_{2\max }$ at baseline (Fig. 1i). The molar sum of plasma BCAAs correlated positively to plasma 3-HIB concentrations (Fig. 1j) and FFM (Fig. 1k). The relationship between the molar sum of plasma BCAAs and liver fat followed a negative exponential distribution (Fig. 1l). We observed no correlation between self-reported protein intake and plasma BCAAs (Fig. 1m). Baseline BCAA level was inversely associated with change in GIR (ρ = −0.41, *p* = 0.037) and $\dot{V}{\mathrm{O}}_{2\max }$ (ρ = −0.47, *p* = 0.015) after 12 weeks of exercise (Fig. 1n, o).

### Exercise

**Fig. 2:**
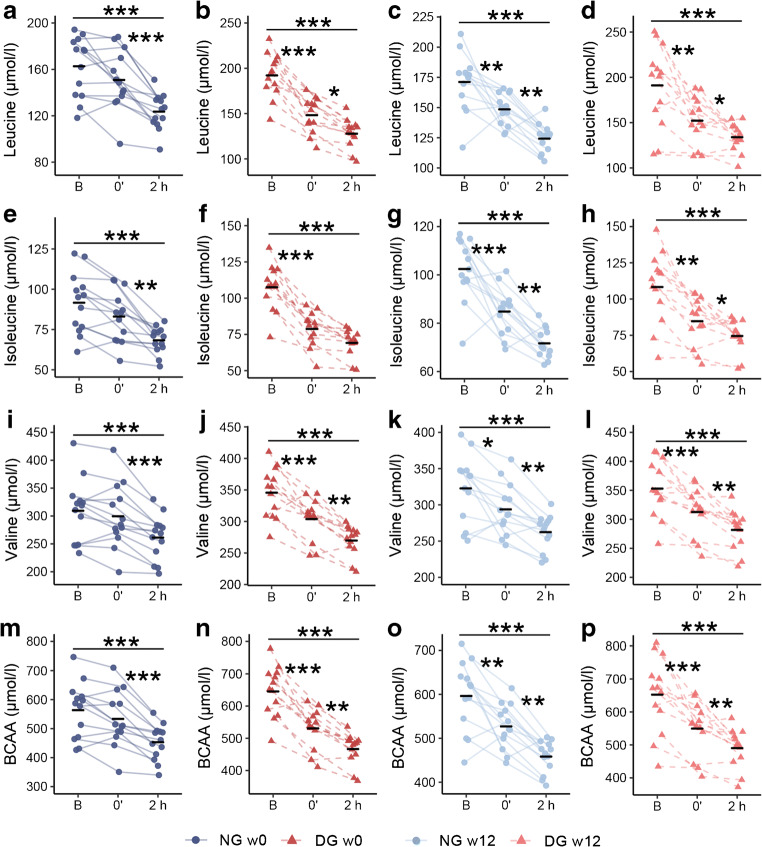
Plasma BCAAs before and after the 45 min bicycle challenge performed before and after 12 weeks of exercise. (**a**–**l**) Concentrations of plasma leucine (**a**–**d**), isoleucine (**e**–**h**) and valine (**i**–**l**), and (**m**–**p**) the molar sum of BCAAs. *p < 0.05, **p < 0.01 and ***p < 0.001; asterisks placed between two time points indicate statistical significance of the response between these two time points; asterisks above the black line indicate the response from before to 2 h after the bicycle challenge. B, before 45 min of cycling; 0′, just after cycling; 2 h, 2 h after cycling; w0, before 12 weeks of exercise intervention; w12, after 12 weeks of exercise intervention

We observed marked reductions in plasma concentrations of leucine (Fig. 2a–d), isoleucine (Fig. 2e–h), valine (Fig. 2i–l), and their molar sum (Fig. 2m–p), as well as of tryptophan (ESM Fig. 2a–d), tyrosine (ESM Fig. 2e–h) and phenylalanine (ESM Fig. 2i–l) both just after, and even more pronounced 2 h after, finishing cycling. These responses were observed for both DG and NG men at both tests (ESM Fig. 2).

Plasma concentration of 3-HIB increased just after cycling and returned to baseline within 2 h for both groups at both bicycle tests (ESM Fig. 2m–p).

The LAT1 transporter consists of two monomers, encoded by *SLC3A2* and *SLC7A5*. At baseline, *SLC3A2* mRNA increased 2 h after cycling whereas *SLC7A5* increased just after and 2 h after the test. The response pattern was similar after the 12 week intervention, except for *SLC3A2* which increased just after and remained elevated 2 h after exercise. There were no differences between groups (ESM Fig. 3a–c).

We observed no change in plasma concentrations of valine, leucine, isoleucine, tryptophan, tyrosine, phenylalanine or 3-HIB after 12 weeks of exercise (Fig. 2), with the exception of increased isoleucine in NG men (10 μmol/l, *p* = 0.01, ‘B’ in Fig. 2e vs ‘B’ in Fig. 2g).

### mRNA-sequencing pathway analysis

**Fig. 3:**
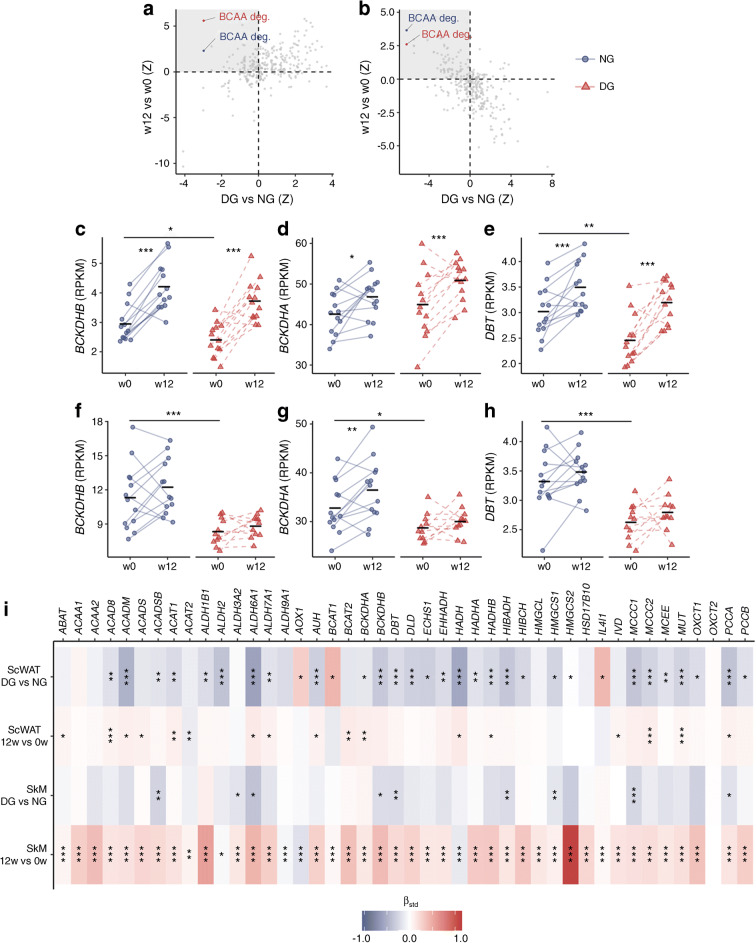
mRNAs of BCAA catabolic genes in SkM and ScWAT. (**a**, **b**) Pathway analysis of SkM (**a**) and ScWAT (**b**) mRNA-sequencing data showing *z* scores for men with DG vs NG at baseline on the *x*-axis, and *z* scores for pathway responses to the intervention in each group (red, DG; blue, NG) on the *y*-axis. The top left quadrant indicates pathways with lower transcript levels in DG vs NG and increased transcript levels after 12 weeks of exercise. Only the BCAA degradation pathway reached significance in all comparisons (details are provided in ESM Table 2). Pathways not reaching the set criteria (ESM Table 2) are presented in grey. (**c**–**h**) mRNA levels for the genes encoding the rate-limiting BCKDH enzyme in SkM (**c**–**e**) and ScWAT (**f**–**h**). *p < 0.05, **p < 0.01 and ***p < 0.001; asterisks placed between two time points indicate statistical significance of the response between these two time points; asterisks above the black line indicate a group difference at baseline. The main effects of 12 weeks of exercise across both groups (week 12 vs week 0) are: (**c**) *p* < 0.001; (**d**) *p* < 0.001; (**e**) *p* < 0.001; (**f**) *p* = 0.057; (**g**) *p* = 0.003; (**h**) *p* = 0.055. (**i**) A response heat map for all BCAA catabolic mRNAs showing the main effects of DG and 12 weeks of exercise in SkM and ScWAT. The colour key indicates standardised β and red indicates genes upregulated and blue downregulated. deg., degradation; w0, before 12 weeks of exercise intervention; w12, after 12 weeks of exercise intervention

BCAA catabolism was the only pathway associated with both dysglycaemia and 12 weeks of exercise in both groups and tissues (ESM Table 2). The BCAA catabolic pathway was lower in DG vs NG men at baseline in SkM (Fig. 3a) and ScWAT (Fig. 3b) and increased after 12 weeks of exercise in both groups in both SkM (Fig. 3a) and ScWAT (Fig. 3b). The response in SkM from DG men was significantly larger than NG men (Fig. 3a and ESM Fig. 4 a) whereas the response in ScWAT was similar in the two groups (Fig. 3b and ESM Fig. 4b). All pathways differing between DG and NG men in SkM and ScWAT at baseline, and all pathway responses to 12 weeks of exercise in both groups and tissues are presented in ESM Tables 3–7. Similar to observations on the pathway level (Fig. 3a, b), median mRNA levels of BCAA catabolic genes increased significantly more in DG than NG men after 12 weeks of exercise in SkM, but not in ScWAT (ESM Fig. 4a, b).

mRNA levels of the three main components of the rate-limiting enzyme BCKDH (encoded by *BCKDHB, BCKDHA* and *DBT*) were lower in men with DG than NG in both SkM (Fig. 3c–e) and ScWAT (Fig. 3f–h) at baseline, with the exception of *BCKDHA* in SkM, which did not differ between groups (Fig. 3d). Transcript levels of SkM *BCKDHB* (Fig. 3c)*, BCKDHA* (Fig. 3d) and *DBT* (Fig. 3e) increased after 12 weeks of exercise in both DG and NG men. In ScWAT, we observed increased mRNA levels of *BCKDHA* after 12 weeks of exercise across both groups (Fig. 3g), reaching significance only in NG men (Fig. 3g). The increased mRNA levels for *BCKDHB* (Fig. 3f) and *DBT* (Fig. 3h) in ScWAT did not reach statistical significance (*p* = 0.057 and *p* = 0.055, respectively). Pairwise results from the mixed model for each mRNA and tissue are presented in ESM Tables 8 and 9.

### Correlations between BCAA catabolic mRNA and phenotypes

**Fig. 4:**
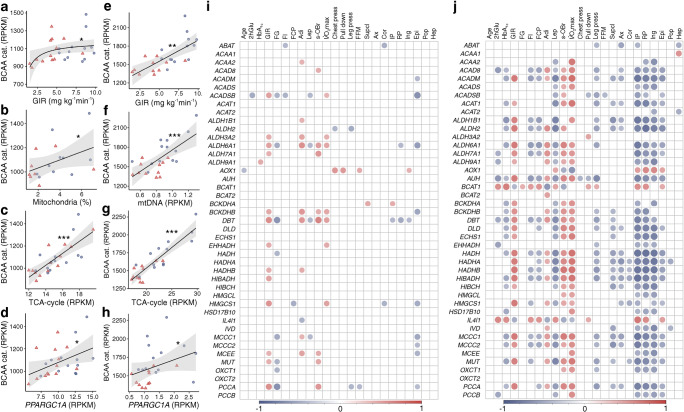
mRNAs of BCAA catabolic genes and correlations with phenotypic traits. Linear regression between median mRNA levels of all BCAA catabolic genes in SkM and (**a**) GIR, (**b**) per cent mitochondria on SkM electron micrographs (only available from 18 participants; see Methods), (**c**) median mRNA levels of all TCA-cycle genes and (**d**) *PPARGC1A* mRNA levels. (**e**–**h**) As for (**a**–**d**), but for ScWAT, except that mitochondrial DNA expression was used as a proxy for mitochondrial content (we have no micrographs of ScWAT). Shaded areas indicate 95% CI of the trend lines. *p < 0.05, **p < 0.01 and ***p < 0.001. (**i**, **j**) Spearman’s correlation heat map between BCAA catabolic mRNAs and relevant phenotypes in SkM (**i**) and ScWAT (**j**). The colour keys indicate Spearman’s ρ. cat., catabolic genes; RPKM, reads per kilobase of transcript per million mapped reads; mtDNA, mitochondrial DNA; 2hGlu, 2 h glucose after an OGTT; F, fasting; G, glucose; I, insulin; CP, C-peptide; Adi, high-molecular mass adiponectin; Lep, leptin; s-OBr, soluble leptin receptor; Supcl, supraclavicular fat; Ax, axillary fat; Cor, cordial fat; IP, intraperitoneal fat; RP, retroperitoneal fat; Ing, inguinal fat; Epi, epididymal fat; Pop, popliteal fat; Hep, hepatic fat

SkM median mRNA levels of all BCAA catabolic genes correlated positively to GIR (Fig. 4a), percentage of mitochondria on SkM micrographs (Fig. 4b), median transcript level of all TCA-cycle genes in (Fig. 4c) and to *PPARGC1A* (Fig. 4d). The results were similar in ScWAT (Fig. 4e–h).

We observed some baseline correlations between individual SkM BCAA catabolic mRNA and phenotypes (Fig. 4i), but these correlations where more pronounced in ScWAT (Fig. 4j). Whereas most transcripts were positively correlated with markers of insulin sensitivity they displayed negative correlations to fat depot sizes (Fig. 4j). These correlations were similar using data after 12 weeks of exercise (ESM Fig. 5). By contrast, the correlations of circulating BCAA with glucometabolic traits and body composition (Fig. 1i) were diminished using data after 12 weeks of exercise (ESM Fig. 6). Results presented in Fig. 1i and ESM Fig. 6 remained similar after correction for FFM (ESM Fig. 7a, b).

### BCKDH kinase and phosphatase and SLC25A44 mitochondrial BCAA transporter mRNA

We observed no differences in *BCKDK, PPM1K* and *SLC25A44* mRNA in SkM and ScWAT between DG and NG men at baseline (ESM Fig. 8), with the exception of ScWAT *SLC25A44* mRNA, which were lower in DG than NG men (ESM Fig. 8f). In response to 12 weeks of exercise, *BCKDK* mRNA remained unaltered in SkM in both groups (ESM Fig. 8a) as well as in ScWAT from NG men (ESM Fig. 8d) but decreased in ScWAT from DG men (ESM Fig. 8d). *PPM1K* mRNA increased in both tissues and both groups (ESM Fig. 8b, e). SkM *SLC25A44* mRNA increased in both groups (ESM Fig. 8c) whereas ScWAT *SLC25A44* mRNA decreased in both groups (ESM Fig. 8f).

### Mediation analysis of BCAA catabolic mRNA on exercise-induced insulin sensitivity

**Fig. 5:**
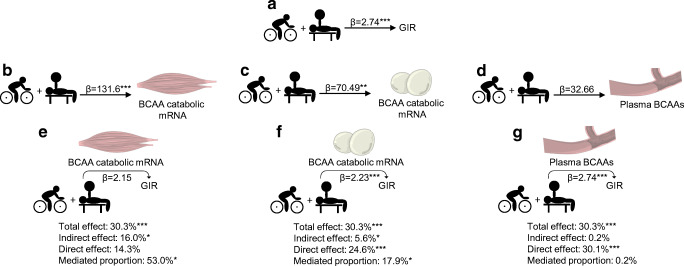
Mediation analyses of SkM, ScWAT, plasma BCAA concentration and insulin sensitivity. (**a**) To test for mediation we constructed a null model for the effect of 12 weeks of exercise on GIR (see Methods). Then we constructed a mediation model in a similar fashion to the null model but substituting GIR with either the median of (**b**) SkM or (**c**) ScWAT BCAA catabolic mRNA levels, or (**d**) the molar sum of plasma BCAAs. (**e**–**g**) Finally, we constructed models similar to the null model but including the potential mediator as a covariate. Analyses were performed on log-transformed data to approximate normality and were back-transformed for presentation. The β value for GIR represents mg kg^−1^ min^−1^ glucose during the clamp, β values for SkM and ScWAT mRNA levels represent reads per kilobase of transcript per million mapped reads (RPKM), and the β value for plasma BCAA level represents μmol/l. Images of muscle, blood vessels and adipocytes are from Servier Medical Art by Servier, which is licensed under a Creative Commons Attribution 3.0 Unported License

We observed that transcript levels of SkM BCAA catabolic mRNA mediated ~53% (*p* = 0.022) of the exercise-induced increase in GIR and that the direct effect of exercise on GIR was no longer statistically significant (*p* = 0.066) after accounting for mediation (Fig. 5e). ScWAT BCAA catabolic mRNA mediated ~18% (*p* = 0.018) of the exercise-induced increase in GIR, but the direct effect of exercise on GIR remained significant after accounting for mediation (Fig. 5f). The partial mediation effect of ScWAT mRNA was no longer significant after adjusting for SkM mRNA (data not shown). Circulating BCAAs did not mediate any effect on exercise-induced increase in GIR (Fig. 5g). The results were similar after correction for age and BMI (ESM Table 10).

## Discussion

Our main findings were that men with dysglycaemia exhibited elevated plasma concentrations of BCAAs and related metabolites compared with men with normoglycaemia. Plasma concentrations of BCAAs and related metabolites correlated positively with markers of liver fat, adiposity and insulin resistance at baseline. Twelve weeks of exercise promoted substantially reduced liver fat and insulin resistance and improved body composition, but circulating BCAAs and related metabolites remained largely unaltered. mRNA-sequencing data implied increased BCAA catabolism in both SkM and ScWAT in both NG and DG men after 12 weeks of exercise. Mediation analyses suggested that the positive effects of exercise on insulin sensitivity might be caused by enhanced BCAA metabolism in SkM and ScWAT.

Several studies reported elevated plasma concentrations of BCAAs and aromatic amino acids in insulin-resistant men and women with [1, 5, 37–40] and without obesity [41]. Several reports indicate a predictive role for plasma BCAAs on insulin resistance and type 2 diabetes [1–9], and a Mendelian randomisation study implied a causal relationship between insulin resistance and BCAA metabolism [42]. Interventions such as gastric bypass surgery [43] and weight loss from energy restriction [2] promoted reduced levels of circulating BCAAs and improved insulin sensitivity [2, 43]. These data have led to the hypothesis of a causal role of disrupted BCAA homeostasis in insulin resistance and type 2 diabetes.

Our data demonstrated higher concentrations of plasma BCAAs and related metabolites in DG compared with NG men at baseline, in line with previous reports [1, 5, 37–41]. Plasma concentrations of BCAAs and aromatic amino acids were highly correlated, as also shown by others [1, 37, 40]. The co-occurrence of BCAAs and aromatic amino acids in plasma has been attributed to their reliance on the same amino acid transporter (LAT1) [44, 45]. We also observed higher plasma concentration of the valine catabolic intermediate 3-HIB in DG men. This may indicate higher catabolism of valine in men with DG, or it could reflect reduced efficiency of the TCA cycle leading to accumulation of BCAA intermediates [19, 46]. In line with previous reports, we showed that not only plasma BCAAs, but also plasma 3-HIB and aromatic amino acids, correlated negatively with markers of insulin sensitivity [37, 40, 41, 47].

We identified that elevated plasma BCAA concentration at baseline correlated with the subsequent change in GIR following 12 weeks of exercise. Thus, based on both our data and previous studies we expected plasma BCAA levels to be reduced after the 12 weeks of exercise. However, plasma BCAA levels did not respond to the intervention, and in fact increased with regard to isoleucine in NG men. Our results are in line with a previous study of nine overweight participants undergoing 6 months of exercise improving insulin sensitivity with no alteration in circulating BCAAs [16]. On the other hand, a recent study found a decrease in plasma BCAAs after 12 weeks of exercise in responders to the intervention [15]. Both our study and the two previous studies looking at the association between BCAAs and insulin resistance after exercise all performed a combined endurance and strength training intervention [15, 16]. It is unlikely that the type of exercise intervention can explain the discrepancy between the studies. The observed non-response in circulating BCAAs after 12 weeks of exercise could be explained by reduced liver fat content [48] and increased BCAA catabolism [21, 49, 50], which would imply a reduction, but also increased FFM, which would imply an increase in plasma BCAA concentrations [51]. In addition, several other factors, such as the balance between protein synthesis and proteolysis in SkM and splanchnic tissue (gastrointestinal tract, liver and other visceral organs) could be influenced by exercise and exhibit effects on circulating BCAAs [52]. Furthermore, gut microbiota [53], genetics [12] and urinary excretion [16] may also influence circulating BCAAs. Dietary factors seemed unrelated to circulating BCAAs in our data, in line with previous studies [1, 54].

Our mRNA-sequencing pathway analysis indicated reduced BCAA catabolism in both SkM and ScWAT from men with DG compared with NG, and increased BCAA catabolism in both groups and tissues after 12 weeks of exercise. We emphasise that these observations are based on mRNA data and we did not measure BCAA catabolism directly. The effect of dysglycaemia on BCAA catabolic mRNA was substantially stronger in ScWAT than SkM, whereas the effect of exercise was substantially stronger in SkM than ScWAT. Moreover, in SkM the effect of exercise on BCAA catabolic mRNA was significantly stronger in DG than in NG men, perhaps promoting normalisation of repressed BCAA catabolism in dysglycaemic SkM. These results are particularly interesting because BCAA catabolism has been suggested as a target for treating insulin resistance [55].

Mediation analyses suggested that the positive effects of exercise on insulin sensitivity are partly promoted by increased BCAA catabolism in SkM and to a lesser degree in ScWAT. However, reverse causality is also possible by increased BCAA catabolic mRNA due to enhanced exercise-induced insulin sensitivity. We also note that mRNA involved in BCAA catabolism cannot mediate changes in insulin sensitivity itself. Future studies should monitor whether proteins involved in BCAA catabolism might mediate changes in insulin sensitivity in SkM. In line with our results on increased BCAA catabolism in tissues after exercise, a recent study showed increased BCAA catabolism in the gut microbiome after 12 weeks of exercise [15]. Our results on the BCAA degradation pathway are also consistent with several previous reports on ScWAT [41, 56–58], as well as lower levels of some transcripts related to BCAA degradation in SkM from patients with type 2 diabetes [59].

The main limitation in our study is the small sample size. The DG group represents a heterogeneous group of men at risk of developing type 2 diabetes, but they also reflect characteristics found widely in society. It is also a shortcoming that no tissue-specific insulin sensitivity was determined, as this could have provided information on the relative contribution of muscle vs hepatic insulin sensitivity to altered BCAA catabolism. In addition, we did not measure turnover of BCAAs directly by, for example, applying stable isotopes to measure BCAA disposal. We note that our results should be interpreted with caution owing to the exploratory nature of our study.

### Conclusion

We showed that plasma concentrations of BCAA and related metabolites were elevated in DG vs NG men and exhibited positive correlations with markers of liver fat, fat mass and insulin resistance. However, plasma concentrations of BCAA and related metabolites were largely unresponsive to 12 weeks of exercise and did not correlate with exercise-induced insulin sensitivity. mRNA-sequencing data implied that SkM and ScWAT BCAA catabolism were increased after 12 weeks of exercise in both groups, and that the insulin-sensitising effect of 12 weeks of exercise could largely be explained by enhanced SkM, and to a lesser degree by enhanced ScWAT, BCAA metabolism.

## Electronic supplementary material

ESM (PDF 1.37 mb)

## Data Availability

The datasets generated during and/or analysed during the current study are available from the corresponding author on reasonable request.

## Abbreviations

BCKDH: Branched-chain α-ketoacid dehydrogenase
BCAA: Branched-chain amino acid
DG: Dysglycaemia (group)
FFM: Fat-free mass
FFQ: Food frequency questionnaire
GIR: Glucose infusion rate
3-HIB: 3-Hydroxyisobutyrate
LAT1: Large amino acid transporter
NG: Normoglycaemia (group)
ScWAT: Subcutaneous white adipose tissue
SkM: Skeletal muscle
TCA: Tricarboxylic acid
