# Supplementary material for: Branched-chain amino acid metabolism, insulin sensitivity and liver fat response to exercise training in sedentary dysglycaemic and normoglycaemic men
Source: Diabetologia. 2020 Oct 29;64(2):410–23. doi: 10.1007/s00125-020-05296-0 (PMC7801320; doi:10.1007/s00125-020-05296-0)
Supplement: Supplementary file 1 — (PDF 1.37 mb) [file 125_2020_5296_MOESM1_ESM.pdf]

ESM Table 1. Protein intake based on food frequency questionnaires before and after 12 weeks of exercise

	**Baseline**				**12 w**				**Change**			
	NG	n	DG	n	NG	n	DG	n	ΔNG	n	ΔDG	n
Protein (g)	108(31)	13	112(44)	12	102(22)	13	108(38)	12	-6.7(31)	13	-1.4(14)	11
Dairy products (g)	592(672)	12	544(367)	12	400(274)	12	519(297)	12	-192(531)	12	-48(192)	11
Energy (MJ)	11702(2643)	13	10990(3279)	12	11586(2502)	13	10443(2932)	13	-116(2168)	13	-81(1812)	12
Milk (g)	558(655)	13	544(367)	12	390(265)	13	519(297)	12	-168(515)	13	-48(192)	11
Milk+cheese (g)	591(652)	13	582(363)	12	429(272)	13	550(292)	12	-162(517)	13	-50(196)	11

g = grams. MJ = mega joules. None of the comparisons were statistically significant (p ranging from 0.30 to 0.97).

ESM Table 2. Selection of the branched-chain amino acids catabolism pathway

	DG vs. NG			NG after 12 weeks		DG after 12 weeks	
Pathway	Score	p-value	FDR	Score	p-value	Score	p-value
<u>ScWAT</u>							
**hsa00280 Valine, leucine and isoleucine degradation**	**-6.2**	**<0.001**	**<0.001**	**2.6**	**0.005**	**3.7**	**<0.001**
hsa00640 Propanoate metabolism	-5.5	<0.001	<0.001	1.9	0.028	2.5	0.008
hsa00020 Citrate cycle (TCA cycle)	-5.5	<0.001	<0.001	3.0	0.002	2.5	0.008
hsa03013 RNA transport	-4.1	<0.001	<0.001	2.9	0.002	-0.6	0.723
hsa00071 Fatty acid metabolism	-3.5	<0.001	0.01	2.0	0.023	2.0	0.023
hsa03040 Spliceosome	-3.4	<0.001	0.01	4.6	<0.001	-0.5	0.682
hsa01040 Biosynthesis of unsaturated fatty acids	-3.3	0.001	0.02	0.0	0.484	-1.2	0.885
hsa00650 Butanoate metabolism	-3.2	0.001	0.02	0.8	0.211	1.9	0.029
hsa04910 Insulin signalling pathway	-2.9	0.002	0.04	1.1	0.144	-0.6	0.723
hsa00620 Pyruvate metabolism	-2.8	0.003	0.05	0.7	0.250	0.6	0.290
<u>SkM</u>							
**hsa00280 Valine, leucine and isoleucine degradation**	**-3.0**	**0.002**	**0.01**	**5.6**	**<0.001**	**2.3**	**0.012**
hsa03013 RNA transport	-3.0	0.001	0.10	-1.7	0.959	-4.2	1.000
hsa03010 Ribosome	-4.1	<0.001	0.10	-8.7	1.000	-10.3	1.000

Pathway analysis was performed using the R package GAGE (generally applicable gene set enrichments) for mRNA sequencing, as described by *Luo et al.* KEGG (Kyoto encyclopaedia of genes and genomes) pathways were tested using the native workflow. First, dysglycaemic (DG) men was compared to normoglycaemic (NG) men at baseline, in both subcutaneous white adipose tissue (ScWAT) and skeletal muscle (SkM). Correction for multiple testing was performed using a false discovery rate (FDR) of 10%. The significant pathways were then tested for responses to 12 weeks of exercise in each group and tissue. TCA = tricarboxylic acids.

ESM Table 3. Pathways differing in ScWAT from DG vs. NG men at baseline

Pathway	Stat.	p-value	FDR (%)
Up regulated in DG vs. NG			
hsa04142 Lysosome	7.6	<0.00001	<1
hsa04145 Phagosome	5.4	<0.00001	<1
hsa04380 Osteoclast differentiation	4.7	<0.00001	<1
hsa04640 Hematopoietic cell lineage	3.9	0.00007	<1
hsa04672 Intestinal immune network for IgA production	3.8	0.00015	<1
hsa00531 Glycosaminoglycan degradation	3.8	0.00025	1
hsa04514 Cell adhesion molecules (CAMs)	3.4	0.00043	1
hsa04610 Complement and coagulation cascades	3.4	0.00052	1
hsa04810 Regulation of actin cytoskeleton	3.1	0.00095	2
hsa00532 Glycosaminoglycan biosynthesis - chondroitin sulphate	3.3	0.00107	2
hsa04612 Antigen processing and presentation	3.0	0.00140	2
hsa00511 Other glycan degradation	3.2	0.00149	2
hsa04512 ECM-receptor interaction	3.0	0.00176	2
hsa04062 Chemokine signalling pathway	2.8	0.00235	3
hsa04621 NOD-like receptor signalling pathway	2.6	0.00535	6
hsa04670 Leukocyte transendothelial migration	2.5	0.00577	6
hsa04620 Toll-like receptor signalling pathway	2.5	0.00636	6
hsa04650 Natural killer cell mediated cytotoxicity	2.4	0.00931	8
hsa04510 Focal adhesion	2.3	0.01175	10
hsa04115 p53 signalling pathway	2.2	0.01370	11
hsa04662 B cell receptor signalling pathway	2.2	0.01555	12
hsa00510 N-Glycan biosynthesis	2.1	0.02111	15
hsa04666 Fc gamma R-mediated phagocytosis	2.0	0.02132	15
hsa04660 T cell receptor signalling pathway	2.0	0.02220	15
hsa04974 Protein digestion and absorption	2.0	0.02337	15
hsa04360 Axon guidance	1.7	0.04234	27
hsa00520 Amino sugar and nucleotide sugar metabolism	1.7	0.04732	29
Down regulated in DG vs. NG			
hsa00280 Valine, leucine and isoleucine degradation	-6.2	<0.00001	<1
hsa00640 Propanoate metabolism	-5.5	<0.00001	<1
hsa00020 Citrate cycle (TCA cycle)	-5.5	<0.00001	<1
hsa03013 RNA transport	-4.1	0.00002	<1
hsa00071 Fatty acid metabolism	-3.5	0.00034	1
hsa03040 Spliceosome	-3.4	0.00039	1
hsa01040 Biosynthesis of unsaturated fatty acids	-3.3	0.00103	2
hsa00650 Butanoate metabolism	-3.2	0.00119	2
hsa04910 Insulin signalling pathway	-2.9	0.00227	4
hsa00620 Pyruvate metabolism	-2.8	0.00287	5
hsa03015 mRNA surveillance pathway	-2.8	0.00312	5
hsa03010 Ribosome	-2.6	0.00520	7
hsa04975 Fat digestion and absorption	-2.6	0.00554	7
hsa03008 Ribosome biogenesis in eukaryotes	-2.5	0.00630	7
hsa00190 Oxidative phosphorylation	-2.5	0.00643	7
hsa04146 Peroxisome	-2.5	0.00650	7
hsa00630 Glyoxylate and dicarboxylate metabolism	-2.6	0.00779	7
hsa00310 Lysine degradation	-2.4	0.00911	8
hsa03320 PPAR signalling pathway	-2.3	0.01092	9

hsa03022 Basal transcription factors	-2.3	0.01359	11
hsa03018 RNA degradation	-2.2	0.01579	12
hsa00380 Tryptophan metabolism	-2.2	0.01674	12
hsa00900 Terpenoid backbone biosynthesis	-2.2	0.01821	13
hsa03060 Protein export	-2.1	0.01916	13
hsa03020 RNA polymerase	-2.1	0.02081	14
hsa00410 beta-Alanine metabolism	-2.0	0.02509	16
hsa00830 Retinol metabolism	-1.9	0.02866	17
hsa00053 Ascorbate and aldarate metabolism	-1.8	0.04035	23
hsa00970 Aminoacyl-tRNA biosynthesis	-1.7	0.04258	24
hsa00591 Linoleic acid metabolism	-1.7	0.04395	24
hsa04120 Ubiquitin mediated proteolysis	-1.7	0.04799	25
hsa00910 Nitrogen metabolism	-1.7	0.04859	25

Pathway analysis was performed using the R package GAGE (generally applicable gene set enrichments) for mRNA sequencing, as described by *Luo et al.* KEGG (Kyoto encyclopaedia of genes and genomes) pathways were tested using the native workflow.

ESM Table 3. Pathways responding to 12 weeks of exercise in ScWAT from NG men

Pathway	Stat.	p-value	FDR (%)
Up regulated after 12 weeks exercise in NG men			
hsa00280 Valine, leucine and isoleucine degradation	3.7	0.00023	2
hsa03010 Ribosome	3.5	0.00028	2
hsa00020 Citrate cycle (TCA cycle)	2.5	0.00766	33
hsa00640 Propanoate metabolism	2.5	0.00817	33
hsa02010 ABC transporters	2.2	0.01390	45
hsa00260 Glycine, serine and threonine metabolism	2.2	0.01724	47
hsa00071 Fatty acid metabolism	2.0	0.02299	54
hsa00650 Butanoate metabolism	1.9	0.02947	60
hsa00380 Tryptophan metabolism	1.8	0.03636	66
hsa00310 Lysine degradation	1.7	0.04780	78
Down regulated after 12 weeks exercise in NG men			
hsa03050 Proteasome	-5.1	<0.00001	<1
hsa04650 Natural killer cell mediated cytotoxicity	-4.5	0.00001	<1
hsa04810 Regulation of actin cytoskeleton	-4.2	0.00002	<1
hsa04660 T cell receptor signalling pathway	-3.8	0.00010	<1
hsa04141 Protein processing in endoplasmic reticulum	-2.8	0.00308	10
hsa04722 Neurotrophin signalling pathway	-2.6	0.00546	15
hsa04662 B cell receptor signalling pathway	-2.5	0.00644	15
hsa04664 Fc epsilon RI signalling pathway	-2.4	0.00811	15
hsa04130 SNARE interactions in vesicular transport	-2.3	0.01088	15
hsa03008 Ribosome biogenesis in eukaryotes	-2.3	0.01225	15
hsa04144 Endocytosis	-2.3	0.01249	15
hsa04010 MAPK signalling pathway	-2.2	0.01283	15
hsa00900 Terpenoid backbone biosynthesis	-2.3	0.01373	15
hsa04110 Cell cycle	-2.2	0.01472	15
hsa04120 Ubiquitin mediated proteolysis	-2.2	0.01501	15
hsa04114 Oocyte meiosis	-2.2	0.01567	15
hsa04510 Focal adhesion	-2.2	0.01580	15
hsa04012 ErbB signalling pathway	-2.0	0.02385	21
hsa04062 Chemokine signalling pathway	-2.0	0.02545	21
hsa04145 Phagosome	-2.0	0.02576	21
hsa04710 Circadian rhythm - mammal	-1.9	0.02959	23
hsa04666 Fc gamma R-mediated phagocytosis	-1.8	0.03433	25
hsa04540 Gap junction	-1.8	0.03841	27

Pathway analysis was performed using the R package GAGE (generally applicable gene set enrichments) for mRNA sequencing, as described by *Luo et al.* KEGG (Kyoto encyclopaedia of genes and genomes) pathways were tested using the native workflow.

ESM Table 4. Pathways responding to 12 weeks of exercise in ScWAT from DG men

Pathway	Stat.	p-value	FDR (%)
Up regulated after 12 weeks exercise in DG men			
hsa03040 Spliceosome	4.6	<0.00001	<1
hsa03013 RNA transport	2.9	0.00196537	12
hsa00020 Citrate cycle (TCA cycle)	3.0	0.00218103	12
hsa00280 Valine, leucine and isoleucine degradation	2.6	0.00547109	18
hsa03008 Ribosome biogenesis in eukaryotes	2.6	0.0056117	18
hsa04146 Peroxisome	2.5	0.0074775	20
hsa03018 RNA degradation	2.3	0.0103225	24
hsa03022 Basal transcription factors	2.3	0.0127502	26
hsa00071 Fatty acid metabolism	2.0	0.02333421	42
hsa04710 Circadian rhythm - mammal	2.0	0.02599038	42
hsa00640 Propanoate metabolism	1.9	0.02824158	42
hsa03015 mRNA surveillance pathway	1.8	0.03546614	48
hsa00982 Drug metabolism - cytochrome P450	1.7	0.04846149	61
Down regulated after 12 weeks exercise in DG men			
hsa04142 Lysosome	-6.6	<0.00001	<1
hsa04145 Phagosome	-5.2	<0.00001	<1
hsa04514 Cell adhesion molecules (CAMs)	-4.8	<0.00001	<1
hsa04650 Natural killer cell mediated cytotoxicity	-4.7	<0.00001	<1
hsa04062 Chemokine signalling pathway	-4.7	<0.00001	<1
hsa04380 Osteoclast differentiation	-4.7	<0.00001	<1
hsa04670 Leukocyte transendothelial migration	-4.3	<0.00001	<1
hsa04810 Regulation of actin cytoskeleton	-4.1	<0.00001	<1
hsa04662 B cell receptor signalling pathway	-3.8	0.00010567	<1
hsa04612 Antigen processing and presentation	-3.7	0.00013445	<1
hsa04640 Hematopoietic cell lineage	-3.7	0.00014427	<1
hsa04666 Fc gamma R-mediated phagocytosis	-3.5	0.00024535	<1
hsa04610 Complement and coagulation cascades	-3.2	0.00080928	1
hsa04672 Intestinal immune network for IgA production	-3.0	0.0015263	2
hsa04971 Gastric acid secretion	-3.0	0.00172366	2
hsa04620 Toll-like receptor signalling pathway	-3.0	0.00172405	2
hsa04510 Focal adhesion	-2.9	0.00181302	2
hsa00531 Glycosaminoglycan degradation	-3.0	0.00257158	2
hsa04660 T cell receptor signalling pathway	-2.8	0.00294535	3
hsa04974 Protein digestion and absorption	-2.7	0.00371292	3
hsa04270 Vascular smooth muscle contraction	-2.7	0.00403517	3
hsa04970 Salivary secretion	-2.3	0.01060714	8
hsa04512 ECM-receptor interaction	-2.3	0.0116506	8
hsa04020 Calcium signalling pathway	-2.3	0.01250048	8
hsa04630 Jak-STAT signalling pathway	-2.2	0.01281644	8
hsa00534 Glycosaminoglycan biosynthesis - heparan sulphate	-2.2	0.01569272	10
hsa00532 Glycosaminoglycan biosynthesis - chondroitin sulphate	-2.2	0.01641244	10
hsa04621 NOD-like receptor signalling pathway	-2.1	0.01895596	11
hsa04916 Melanogenesis	-2.1	0.01934421	11
hsa04540 Gap junction	-2.1	0.01979636	11
hsa00510 N-Glycan biosynthesis	-2.1	0.02122177	11
hsa04966 Collecting duct acid secretion	-2.1	0.02242856	11
hsa00052 Galactose metabolism	-2.0	0.02396158	11

hsa04742 Taste transduction	-2.0	0.02419678	11
hsa04623 Cytosolic DNA-sensing pathway	-2.0	0.02427151	11
hsa00511 Other glycan degradation	-1.9	0.03247301	15
hsa00600 Sphingolipid metabolism	-1.9	0.03363006	15
hsa04141 Protein processing in endoplasmic reticulum	-1.8	0.03435275	15
hsa04210 Apoptosis	-1.8	0.03668649	15
hsa00604 Glycosphingolipid biosynthesis - ganglio series	-1.8	0.0382418	16
hsa04914 Progesterone-mediated oocyte maturation	-1.7	0.04270899	17
hsa04972 Pancreatic secretion	-1.7	0.04694591	18

Pathway analysis was performed using the R package GAGE (generally applicable gene set enrichments) for mRNA sequencing, as described by *Luo et al.* KEGG (Kyoto encyclopaedia of genes and genomes) pathways were tested using the native workflow.

ESM Table 5. Pathways differing in SkM from DG vs. NG men at baseline

Pathway	Stat.	p-value	FDR (%)
Up regulated in DG			
hsa03050 Proteasome	3.7	0.0001936	3
hsa04620 Toll-like receptor signalling pathway	3.4	0.00036915	3
hsa04145 Phagosome	3.3	0.00059427	3
hsa04380 Osteoclast differentiation	3.2	0.00081191	3
hsa04650 Natural killer cell mediated cytotoxicity	3.1	0.00108775	4
hsa04666 Fc gamma R-mediated phagocytosis	2.9	0.00191481	5
hsa04514 Cell adhesion molecules (CAMs)	2.8	0.00252415	5
hsa04062 Chemokine signalling pathway	2.8	0.00263607	5
hsa04012 ErbB signalling pathway	2.8	0.00319422	5
hsa04672 Intestinal immune network for IgA production	2.8	0.00335087	5
hsa04662 B cell receptor signalling pathway	2.6	0.00456762	7
hsa04512 ECM-receptor interaction	2.6	0.00511701	7
hsa04510 Focal adhesion	2.5	0.00640457	8
hsa00532 Glycosaminoglycan biosynthesis - chondroitin sulphate	2.6	0.00733605	8
hsa00533 Glycosaminoglycan biosynthesis - keratan sulphate	2.4	0.01269971	14
hsa00051 Fructose and mannose metabolism	2.2	0.01384698	14
hsa04640 Hematopoietic cell lineage	2.2	0.01503084	14
hsa04010 MAPK signalling pathway	2.2	0.01532486	14
hsa04144 Endocytosis	2.1	0.0163216	14
hsa04722 Neurotrophin signalling pathway	2.1	0.01721876	14
hsa00052 Galactose metabolism	2.1	0.01909601	14
hsa04916 Melanogenesis	2.1	0.01924291	14
hsa04810 Regulation of actin cytoskeleton	2.0	0.02302438	16
hsa00010 Glycolysis / Gluconeogenesis	2.0	0.02304336	16
hsa04142 Lysosome	2.0	0.02527717	16
hsa00514 Other types of O-glycan biosynthesis	2.0	0.02589616	16
hsa04612 Antigen processing and presentation	1.9	0.02853203	17
hsa04974 Protein digestion and absorption	1.9	0.03317248	19
hsa00534 Glycosaminoglycan biosynthesis - heparan sulphate	1.8	0.03851783	22
hsa00500 Starch and sucrose metabolism	1.8	0.04011297	22
hsa04664 Fc epsilon RI signalling pathway	1.7	0.0423998	22
hsa04310 Wnt signalling pathway	1.7	0.04306186	22
hsa04660 T cell receptor signalling pathway	1.7	0.04613931	23
Down regulated in DG			
hsa03010 Ribosome	-4.1	<0.00001	1
hsa03013 RNA transport	-3.0	0.00148431	10
hsa00280 Valine, leucine and isoleucine degradation	-3.0	0.00190014	10
hsa00640 Propanoate metabolism	-2.5	0.00676891	27
hsa03015 mRNA surveillance pathway	-2.0	0.02453685	79
hsa03018 RNA degradation	-1.9	0.03060771	79
hsa03060 Protein export	-1.8	0.03816112	79

Pathway analysis was performed using the R package GAGE (generally applicable gene set enrichments) for mRNA sequencing, as described by *Luo et al.* KEGG (Kyoto encyclopaedia of genes and genomes) pathways were tested using the native workflow.

ESM Table 6. Pathways responding to 12 weeks of exercise in SkM from NG men

Pathway	Stat.	p-value	FDR (%)
Up regulated in NG men after 12 weeks of exercise			
hsa04510 Focal adhesion	5.8	<0.00001	<1
hsa04512 ECM-receptor interaction	5.6	<0.00001	<1
hsa04514 Cell adhesion molecules (CAMs)	5.4	<0.00001	<1
hsa04666 Fc gamma R-mediated phagocytosis	4.9	<0.00001	<1
hsa04062 Chemokine signalling pathway	4.8	<0.00001	<1
hsa04145 Phagosome	4.7	<0.00001	<1
hsa04144 Endocytosis	4.4	0.00001	<1
hsa04810 Regulation of actin cytoskeleton	4.4	0.00001	<1
hsa04670 Leukocyte transendothelial migration	4.1	0.00002	<1
hsa04142 Lysosome	3.9	0.00007	<1
hsa04380 Osteoclast differentiation	3.4	0.00043	1
hsa04640 Hematopoietic cell lineage	3.4	0.00043	1
hsa04210 Apoptosis	3.2	0.00077	1
hsa04360 Axon guidance	3.2	0.00077	1
hsa00531 Glycosaminoglycan degradation	3.4	0.00085	1
hsa04520 Adherens junction	3.2	0.00087	1
hsa04612 Antigen processing and presentation	3.1	0.00122	1
hsa04916 Melanogenesis	2.9	0.00190	2
hsa04115 p53 signalling pathway	2.8	0.00316	3
hsa04620 Toll-like receptor signalling pathway	2.7	0.00340	3
hsa04974 Protein digestion and absorption	2.7	0.00427	3
hsa04530 Tight junction	2.6	0.00445	3
hsa04540 Gap junction	2.6	0.00450	3
hsa04672 Intestinal immune network for IgA production	2.7	0.00457	3
hsa00532 Glycosaminoglycan biosynthesis - chondroitin sulphate	2.7	0.00461	3
hsa04664 Fc epsilon RI signalling pathway	2.6	0.00521	3
hsa04270 Vascular smooth muscle contraction	2.5	0.00577	3
hsa04330 Notch signalling pathway	2.5	0.00638	4
hsa04914 Progesterone-mediated oocyte maturation	2.5	0.00647	4
hsa04610 Complement and coagulation cascades	2.5	0.00737	4
hsa04340 Hedgehog signalling pathway	2.4	0.00810	4
hsa04650 Natural killer cell mediated cytotoxicity	2.4	0.00830	4
hsa01040 Biosynthesis of unsaturated fatty acids	2.5	0.00844	4
hsa04070 Phosphatidylinositol signalling system	2.4	0.00923	4
hsa00280 Valine, leucine and isoleucine degradation	2.3	0.01173	5
hsa04310 Wnt signalling pathway	2.3	0.01212	5
hsa04010 MAPK signalling pathway	2.2	0.01319	6
hsa00562 Inositol phosphate metabolism	2.2	0.01403	6
hsa04662 B cell receptor signalling pathway	2.1	0.01981	8
hsa04110 Cell cycle	2.0	0.02402	9
hsa00534 Glycosaminoglycan biosynthesis - heparan sulphate	2.0	0.02405	9
hsa04730 Long-term depression	2.0	0.02459	9
hsa04630 Jak-STAT signalling pathway	1.9	0.02678	10
hsa00600 Sphingolipid metabolism	2.0	0.02741	10
hsa00511 Other glycan degradation	2.0	0.02824	10
hsa04320 Dorso-ventral axis formation	1.8	0.03914	14
hsa04722 Neurotrophin signalling pathway	1.7	0.04925	17

Down regulated in NG men after 12 weeks of exercise			
hsa03010 Ribosome	-10.3	<0.00001	<1
hsa04740 Olfactory transduction	-5.3	<0.00001	<1
hsa03040 Spliceosome	-4.8	<0.00001	<1
hsa03013 RNA transport	-4.2	0.00002	<1
hsa03008 Ribosome biogenesis in eukaryotes	-2.7	0.00446	14
hsa00830 Retinol metabolism	-2.6	0.00591	16
hsa03022 Basal transcription factors	-2.1	0.01838	43

Pathway analysis was performed using the R package GAGE (generally applicable gene set enrichments) for mRNA sequencing, as described by *Luo et al.* KEGG (Kyoto encyclopaedia of genes and genomes) pathways were tested using the native workflow.

ESM Table 7. Pathways responding to 12 weeks of exercise in SkM from DG men

Pathway	Stat.	p-value	FDR (%)
Up regulated in DG men after 12 weeks of exercise			
hsa00190 Oxidative phosphorylation	6.0	<0.00001	<1
hsa00280 Valine, leucine and isoleucine degradation	5.6	<0.00001	<1
hsa00020 Citrate cycle (TCA cycle)	5.1	<0.00001	<1
hsa04512 ECM-receptor interaction	4.5	0.00001	<1
hsa04510 Focal adhesion	4.0	0.00004	<1
hsa04145 Phagosome	3.2	0.00077	2
hsa04670 Leukocyte transendothelial migration	3.1	0.00111	2
hsa04810 Regulation of actin cytoskeleton	3.0	0.00127	2
hsa04666 Fc gamma R-mediated phagocytosis	3.0	0.00133	2
hsa04144 Endocytosis	2.9	0.00199	3
hsa04146 Peroxisome	2.9	0.00217	3
hsa00630 Glyoxylate and dicarboxylate metabolism	3.0	0.00299	4
hsa00640 Propanoate metabolism	2.8	0.00360	4
hsa01040 Biosynthesis of unsaturated fatty acids	2.7	0.00475	5
hsa04270 Vascular smooth muscle contraction	2.6	0.00568	6
hsa00970 Aminoacyl-tRNA biosynthesis	2.5	0.00689	7
hsa00620 Pyruvate metabolism	2.5	0.00724	7
hsa04210 Apoptosis	2.4	0.00894	8
hsa04115 p53 signalling pathway	2.3	0.01084	9
hsa04514 Cell adhesion molecules (CAMs)	2.3	0.01089	9
hsa00290 Valine, leucine and isoleucine biosynthesis	2.4	0.01334	10
hsa04530 Tight junction	2.1	0.01958	14
hsa04962 Vasopressin-regulated water reabsorption	2.1	0.02123	15
hsa04062 Chemokine signalling pathway	2.0	0.02172	15
hsa04330 Notch signalling pathway	2.0	0.02483	16
hsa00561 Glycerolipid metabolism	1.9	0.02873	18
hsa04520 Adherens junction	1.8	0.03453	20
hsa00410 beta-Alanine metabolism	1.9	0.03472	20
hsa00860 Porphyrin and chlorophyll metabolism	1.8	0.03654	20
hsa04730 Long-term depression	1.8	0.04032	22
hsa04974 Protein digestion and absorption	1.7	0.04193	22
hsa04360 Axon guidance	1.7	0.04286	22
hsa00562 Inositol phosphate metabolism	1.7	0.04713	23
Down regulated in DG men after 12 weeks of exercise			
hsa03010 Ribosome	-8.7	<0.00001	<1
hsa04740 Olfactory transduction	-4.3	0.00001	<1
hsa03040 Spliceosome	-3.0	0.00165	9
hsa04710 Circadian rhythm - mammal	-1.8	0.03675	88
hsa00830 Retinol metabolism	-1.8	0.04026	88
hsa03008 Ribosome biogenesis in eukaryotes	-1.8	0.04072	88
hsa03013 RNA transport	-1.7	0.04122	88

Pathway analysis was performed using the R package GAGE (generally applicable gene set enrichments) for mRNA sequencing, as described by *Luo et al.* KEGG (Kyoto encyclopaedia of genes and genomes) pathways were tested using the native workflow.

ESM Table 8. Skeletal muscle branched-chain amino acids catabolic mRNA

Gene	DG vs. NG 0w	NG after 12 weeks	DG after 12 weeks	DG vs. NG 12w
ABAT	-0.051[-0.13,0.025]	0.050[-0.008,0.11]	0.058[-0.001,0.12]	-0.044[-0.12,0.033]
ACAA1	0.39[-2.2,3.0]	4.5[2.9,6.1]***	5.5[3.9,7.1]***	1.4[-1.2,4.0]
ACAA2	-5.3[-13,2.7]	12[7.4,17]***	17[12,22]***	-0.21[-8.1,7.7]
ACAD8	-1.4[-3.2,0.32]	1.4[0.25,2.5]*	2.4[1.3,3.5]***	-0.39[-2.1,1.4]
ACADM	-20[-43,3.7]	5.9[-5.9,18]	22[10,34]***	-3.6[-27,20]
ACADS	0.70[-7.1,8.5]	7.1[2.3,12]**	14[8.8,18]***	7.2[-0.62,15]
ACADSB	-3.6[-5.9,-1.3]**	1.4[-0.21,3.0]	3.8[2.2,5.4]***	-1.2[-3.5,1.1]
ACAT1	0.082[-26,26]	4.0[-9.5,18]	31[17,44]***	27[0.45,53]*
ACAT2	-0.17[-0.45,0.11]	0.10[-0.12,0.33]	0.12[-0.100,0.35]	-0.15[-0.43,0.13]
ALDH1B1	0.95[-5.8,7.7]	9.3[5.1,14]***	11[6.3,15]***	2.1[-4.6,8.9]
ALDH2	-7.2[-18,3.1]	-8.5[-15,-2.1]**	3.5[-2.9,9.9]	4.8[-5.6,15]
ALDH3A2	-1.8[-3.3,-0.24]*	0.60[-0.40,1.6]	2.1[1.1,3.1]***	-0.23[-1.8,1.3]
ALDH6A1	-2.1[-4.5,0.27]	2.2[0.54,3.9]**	3.7[2.0,5.4]***	-0.65[-3.1,1.8]
ALDH7A1	-0.39[-1.1,0.30]	0.72[0.33,1.1]***	1.1[0.72,1.5]***	-0.002[-0.69,0.69]
ALDH9A1	-0.22[-3.7,3.3]	-2.5[-4.5,-0.45]*	-1.9[-3.9,0.088]	0.31[-3.2,3.8]
AOX1	-0.055[-0.29,0.18]	-0.12[-0.30,0.054]	-0.19[-0.36,-0.012]*	-0.12[-0.36,0.12]
AUH	-0.79[-2.4,0.83]	1.6[0.53,2.7]**	3.3[2.2,4.3]***	0.87[-0.75,2.5]
BCAT1	-0.009[-0.056,0.037]	0.057[0.014,0.099]**	0.037[-0.005,0.079]	-0.029[-0.075,0.018]
BCAT2	0.33[-1.3,1.9]	2.8[1.9,3.7]***	3.4[2.5,4.3]***	0.97[-0.62,2.6]
BCKDHA	2.4[-2.5,7.2]	4.3[0.83,7.7]*	6.0[2.5,9.4]***	4.1[-0.83,8.9]
BCKDHB	-0.54[-1.1,0.013]	1.3[0.87,1.6]***	1.3[0.93,1.7]***	-0.49[-1.0,0.067]
DBT	-0.56[-0.92,-0.21]**	0.48[0.23,0.72]***	0.74[0.49,0.98]***	-0.30[-0.66,0.057]
DLD	-6.9[-17,2.9]	9.7[4.1,15]***	17[12,23]***	0.58[-9.3,10]
ECHS1	-7.9[-19,3.2]	6.1[0.26,12]*	16[10,22]***	2.0[-9.1,13]
EHHADH	-0.24[-0.49,0.010]	0.082[-0.055,0.22]	0.32[0.19,0.46]***	0.003[-0.25,0.25]
HADH	-4.5[-15,6.3]	-12[-18,-5.4]***	-6.2[-13,0.14]	1.0[-9.9,12]
HADHA	-2.6[-21,16]	24[12,37]***	38[26,51]***	12[-6.9,30]
HADHB	-33[-86,19]	42[9.4,75]*	74[41,107]***	-1.9[-54,51]
HIBADH	-7.5[-13,-2.1]**	1.8[-0.83,4.5]	7.1[4.4,9.7]***	-2.3[-7.7,3.1]
HIBCH	0.37[-3.7,4.4]	4.6[2.3,6.9]***	5.9[3.6,8.2]***	1.7[-2.4,5.7]
HMGCL	0.011[-2.9,2.9]	0.66[-1.1,2.5]	3.2[1.4,5.0]***	2.5[-0.35,5.4]
HMGCS1	-0.77[-1.3,-0.20]**	0.040[-0.39,0.47]	0.28[-0.15,0.71]	-0.53[-1.1,0.038]
HSD17B10	-0.37[-3.2,2.4]	4.3[2.3,6.3]***	6.5[4.5,8.5]***	1.8[-0.97,4.6]
IL4I1	0.041[-0.089,0.17]	0.014[-0.046,0.074]	0.016[-0.044,0.076]	0.043[-0.088,0.17]
IVD	-0.16[-2.5,2.1]	0.61[-0.44,1.6]	2.7[1.7,3.8]***	1.9[-0.35,4.2]
MCCC1	-1.9[-3.0,-0.77]***	1.1[0.47,1.8]***	1.7[0.99,2.3]***	-1.3[-2.4,-0.25]*
MCCC2	-1.0[-2.9,0.87]	1.3[0.18,2.4]*	2.6[1.5,3.8]***	0.31[-1.6,2.2]
MCEE	-1.9[-4.2,0.28]	1.00[-0.51,2.5]	2.2[0.68,3.7]**	-0.75[-3.0,1.5]
MUT	-1.2[-2.5,-0.027]*	0.93[0.28,1.6]**	1.7[1.0,2.3]***	-0.52[-1.7,0.70]
OXCT1	-1.3[-3.6,0.95]	1.0[-0.23,2.3]	1.4[0.13,2.7]*	-0.97[-3.3,1.3]
OXCT2	0.003[-0.071,0.078]	0.063[-0.004,0.13]	0.010[-0.056,0.077]	-0.050[-0.12,0.025]
PCCA	-0.81[-1.5,-0.15]*	0.24[-0.17,0.64]	0.88[0.47,1.3]***	-0.17[-0.83,0.49]
PCCB	-1.3[-6.3,3.6]	4.9[2.2,7.5]***	6.6[3.9,9.2]***	0.37[-4.6,5.3]

Results from the mixed model constructed using the R package *lme4.* The contrasts shown were computed using the R package *emmeans.* Beta values with 95 % confidence intervals are shown and represents reads per kilobase of transcript per million mapped reads (RPKM). ***p<0.001, **p<0.01 and *p<0.05. "DG vs. NG 0w" = comparison of dysglycaemic (DG) vs. normoglycaemic (NG) men at baseline. "NG after 12 weeks" and "DG after 12 weeks" = response to 12 weeks of exercise within each group. "DG vs. NG 12w" = comparison of DG vs. NG men after 12 weeks of exercise.

ESM Table 9. Subcutaneous white adipose tissue branched-chain amino acids catabolic mRNA

Gene	DG vs. NG 0w	NG after 12 weeks	DG after 12 weeks	DG vs. NG 12w
ABAT	-0.078[-0.19,0.032]	0.065[-0.012,0.14]	0.063[-0.014,0.14]	-0.080[-0.19,0.030]
ACAA1	0.062[-0.051,0.17]	0.017[-0.057,0.092]	0.022[-0.052,0.096]	0.066[-0.046,0.18]
ACAA2	-0.081[-0.23,0.063]	0.040[-0.040,0.12]	0.027[-0.053,0.11]	-0.095[-0.24,0.050]
ACAD8	-0.18[-0.29,-0.074]**	0.083[0.025,0.14]**	0.055[-0.003,0.11]	-0.21[-0.32,-0.10]***
ACADM	-0.48[-0.71,-0.25]***	0.089[-0.038,0.22]	0.099[-0.027,0.23]	-0.47[-0.70,-0.24]***
ACADS	-0.15[-0.32,0.025]	0.094[-0.010,0.20]	0.072[-0.031,0.18]	-0.17[-0.34,0.004]
ACADSB	-0.17[-0.29,-0.046]**	0.031[-0.055,0.12]	0.062[-0.024,0.15]	-0.14[-0.26,-0.015]*
ACAT1	-0.23[-0.38,-0.074]**	0.13[0.049,0.20]**	0.053[-0.023,0.13]	-0.30[-0.45,-0.15]***
ACAT2	-0.11[-0.28,0.062]	-0.13[-0.24,-0.019]*	-0.11[-0.22,0.002]	-0.089[-0.26,0.082]
ALDH1B1	-0.22[-0.35,-0.082]**	0.075[-0.031,0.18]	0.018[-0.088,0.13]	-0.27[-0.41,-0.14]***
ALDH2	-0.38[-0.57,-0.19]***	-0.002[-0.12,0.12]	0.091[-0.027,0.21]	-0.28[-0.47,-0.096]**
ALDH3A2	-0.023[-0.14,0.092]	-0.084[-0.16,-0.009]*	0.019[-0.057,0.094]	0.079[-0.035,0.19]
ALDH6A1	-0.59[-0.89,-0.28]***	0.23[0.050,0.41]*	0.028[-0.15,0.21]	-0.79[-1.1,-0.49]***
ALDH7A1	-0.17[-0.28,-0.065]**	0.059[-0.025,0.14]	0.088[0.005,0.17]*	-0.14[-0.25,-0.036]**
ALDH9A1	-0.22[-0.48,0.039]	-0.007[-0.18,0.17]	0.045[-0.13,0.22]	-0.17[-0.43,0.092]
AOX1	0.28[0.049,0.52]*	-0.044[-0.20,0.11]	-0.033[-0.19,0.12]	0.29[0.059,0.53]*
AUH	-0.30[-0.43,-0.16]***	0.10[0.002,0.20]*	0.045[-0.057,0.15]	-0.36[-0.49,-0.22]***
BCAT1	0.43[0.071,0.78]*	-0.016[-0.27,0.24]	-0.25[-0.50,0.010]	0.20[-0.16,0.55]
BCAT2	-0.16[-0.34,0.025]	0.16[0.058,0.27]**	0.079[-0.026,0.18]	-0.24[-0.42,-0.059]**
BCKDHA	-0.15[-0.29,-0.005]*	0.15[0.048,0.25]**	0.053[-0.047,0.15]	-0.24[-0.39,-0.100]***
BCKDHB	-0.33[-0.52,-0.14]***	0.12[-0.022,0.26]	0.065[-0.076,0.20]	-0.38[-0.57,-0.20]***
DBT	-0.22[-0.33,-0.11]***	0.059[-0.029,0.15]	0.056[-0.032,0.14]	-0.22[-0.33,-0.12]***
DLD	-0.29[-0.46,-0.12]***	0.049[-0.045,0.14]	-0.020[-0.11,0.075]	-0.36[-0.53,-0.19]***
ECHS1	-0.30[-0.54,-0.066]*	0.038[-0.10,0.18]	0.036[-0.10,0.18]	-0.30[-0.54,-0.067]*
EHHADH	-0.22[-0.38,-0.059]**	0.071[-0.046,0.19]	0.075[-0.042,0.19]	-0.22[-0.38,-0.055]**
HADH	-0.55[-0.81,-0.30]***	0.14[-0.001,0.28]	0.11[-0.029,0.25]	-0.58[-0.84,-0.32]***
HADHA	-0.17[-0.29,-0.060]**	0.046[-0.003,0.095]	0.009[-0.040,0.058]	-0.21[-0.32,-0.098]***
HADHB	-0.24[-0.36,-0.11]***	0.056[-0.004,0.12]	0.034[-0.026,0.093]	-0.26[-0.39,-0.13]***
HIBADH	-0.34[-0.50,-0.19]***	0.086[-0.008,0.18]	0.006[-0.088,0.10]	-0.42[-0.58,-0.27]***
HIBCH	-0.24[-0.44,-0.041]*	0.028[-0.11,0.16]	0.024[-0.11,0.16]	-0.24[-0.44,-0.045]*
HMGCL	-0.076[-0.16,0.004]	0.013[-0.060,0.085]	0.016[-0.057,0.089]	-0.073[-0.15,0.007]
HMGCS1	-0.26[-0.50,-0.023]*	-0.093[-0.21,0.025]	-0.018[-0.14,0.10]	-0.19[-0.43,0.053]
HMGCS2	-0.004[-0.008,0.000]*	-0.002[-0.006,0.002]	0.000[-0.004,0.004]	-0.002[-0.007,0.002]
HSD17B10	-0.090[-0.23,0.051]	-0.018[-0.098,0.063]	0.016[-0.065,0.096]	-0.057[-0.20,0.085]
IL4I1	0.41[0.077,0.75]*	-0.029[-0.35,0.29]	-0.14[-0.46,0.18]	0.30[-0.034,0.64]
IVD	-0.11[-0.26,0.049]	0.088[0.003,0.17]*	0.041[-0.044,0.13]	-0.15[-0.31,0.003]
MCCC1	-0.36[-0.56,-0.15]***	0.15[0.021,0.28]*	0.027[-0.10,0.16]	-0.48[-0.69,-0.28]***
MCCC2	-0.26[-0.40,-0.13]***	0.097[0.013,0.18]*	0.11[0.023,0.19]*	-0.25[-0.39,-0.12]***
MCEE	-0.18[-0.30,-0.058]**	0.015[-0.082,0.11]	0.009[-0.088,0.11]	-0.18[-0.30,-0.064]**
MUT	-0.27[-0.40,-0.13]***	0.051[0.001,0.10]*	0.069[0.019,0.12]**	-0.25[-0.38,-0.11]***
OXCT1	-0.22[-0.42,-0.011]*	0.055[-0.083,0.19]	0.030[-0.11,0.17]	-0.24[-0.45,-0.036]*
OXCT2	0.017[-0.002,0.037]	0.030[0.011,0.048]**	-0.003[-0.022,0.015]	-0.016[-0.035,0.004]
PCCA	-0.38[-0.57,-0.18]***	0.12[0.029,0.20]**	0.041[-0.046,0.13]	-0.45[-0.65,-0.26]***
PCCB	-0.19[-0.35,-0.022]*	0.079[-0.023,0.18]	0.054[-0.048,0.16]	-0.21[-0.38,-0.046]*

Results from the mixed model constructed using the R package *lme4.* The contrasts shown were computed using the R package *emmeans.* Beta values with 95 % confidence intervals are shown and represents reads per kilobase of transcript per million mapped reads (RPKM). ***p<0.001, **p<0.01 and *p<0.05. "DG vs. NG 0w" = comparison of dysglycaemic (DG) vs. normoglycaemic (NG) men at baseline. "NG after 12 weeks" and "DG after 12 weeks" = response to 12 weeks of exercise within each group. "DG vs. NG 12w" = comparison of DG vs. NG men after 12 weeks of exercise.

ESM Table 10. Mediation analyses corrected for BMI and age.

SkM

BMI

	Beta	95%CI low	95%CI high	p-value
ACME	0.1793	0.0389	0.32	0.014
ADE	0.1188	0.0617	0.28	0.184
Total effect	0.2981	0.1895	0.40	<2e-16
Prop. mediated	0.6041	0.1373	1.30	0.014

Age

	Beta	95%CI low	95%CI high	p-value
ACME	0.146843	0.026950	0.28	0.010
ADE	0.154185	0.000307	0.31	0.048
Total effect	0.301028	0.188730	0.41	<2e-16
Prop. mediated	0.487639	0.081106	1.00	0.010

ScWAT

BMI

	Beta	95%CI low	95%CI high	p-value
ACME	0.05325	0.00904	0.12	0.016
ADE	0.24635	0.12765	0.37	<2e-16
Total effect	0.29959	0.17596	0.42	<2e-16
Prop. mediated	0.16935	0.03336	0.41	0.016

Age

	Beta	95%CI low	95%CI high	p-value
ACME	0.05856	0.00741	0.13	0.016
ADE	0.24824	0.13313	0.36	<2e-16
Total effect	0.30680	0.19571	0.42	<2e-16
Prop. mediated	0.18306	0.02728	0.42	0.016

ACME; average causal mediation effect. ADE; average direct effect. Prop.; proportion. CI; confidence interval.

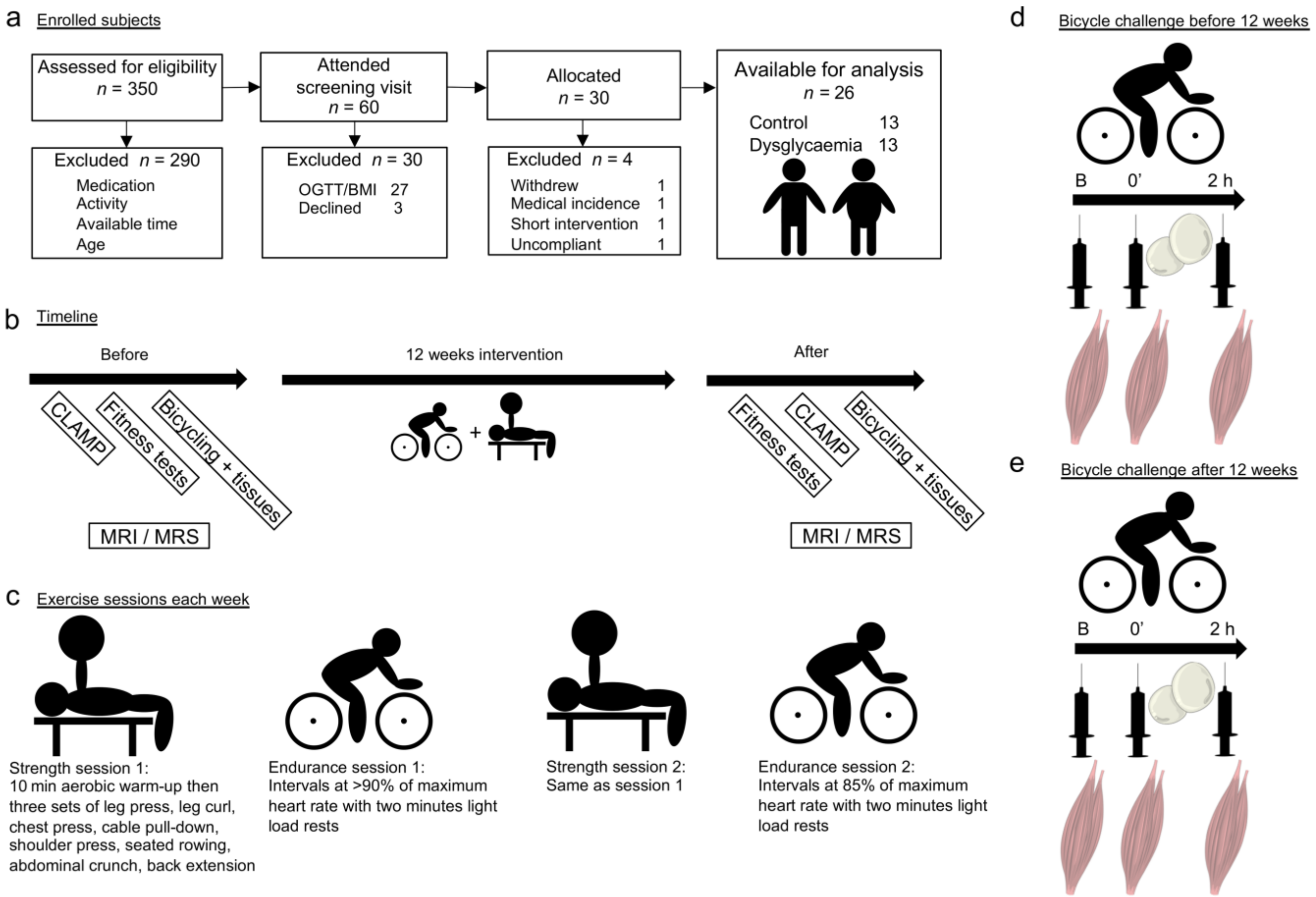

ESM Fig. 1. Study design. (A) Twenty-six sedentary men with or without overweight and dysglycaemia were recruited into two groups. (B) The participants donated tissue samples, and underwent several tests at baseline, including hyperinsulinaemic euglycaemic clamp before being subjected to 12 weeks of intensive physical exercise intervention followed by re-testing. Three days passed between the last bout of exercise and the hyperinsulinaemic euglycaemic clamp after intervention. (C) The intervention consisted of four exercise sessions each week. (D) Blood and muscle were harvested before, just after and 2 hours after a 45 min bicycle challenge at 70 % VO_2max before, and (E) after the 12 weeks intervention. Adipose tissue was obtained just after the bicycle challenges (two subjects did not donate adipose tissue). MRI/MRS, magnetic resonance imaging/spectrometry. © Springer Nature, reproduced from [22] with permission. Images of muscle cells and adipocytes are from Servier Medical Art by Servier, which is licensed under a Creative Commons Attribution 3.0 Unported License

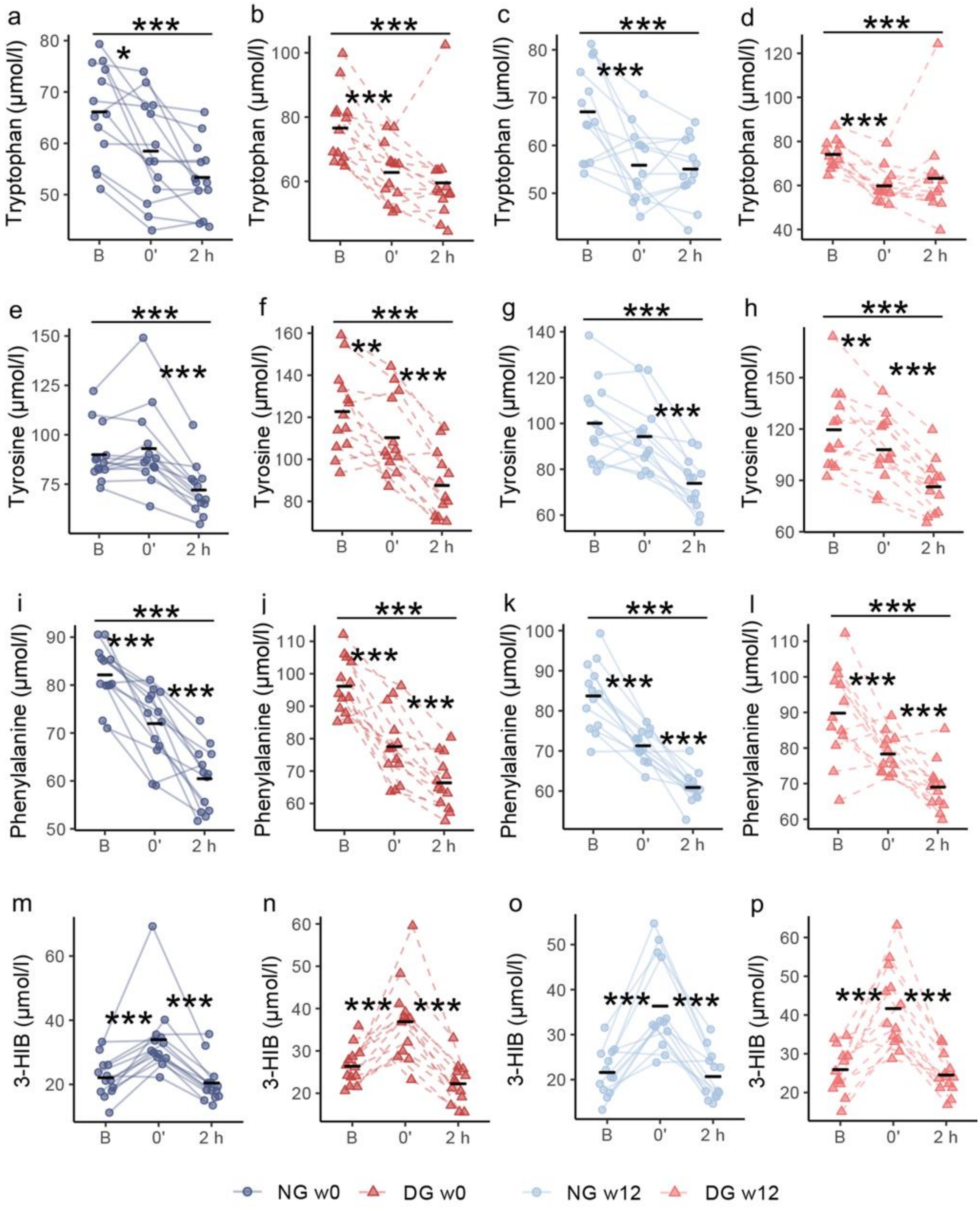

ESM Fig. 2. Plasma aromatic amino acids and 3-hydroxy-isobutyrate (3-HIB). The legend below the figure applies to all panels and shows that normoglycaemic (NG) men are presented in blue and dysglycaemic (DG) men in red. Dark colours represent the bicycle challenge performed before 12 weeks of exercise and light colours represent the bicycle challenge performed after 12 weeks of exercise. (a-d) Concentrations of plasma tryptophan, (e-h) tyrosine, (i-l) phenylalanine and (m-p) 3-HIB. *p<0.05, **p<0.01 and ***p<0.001. The significance star(s) placed between two time points indicates statistical significance of the response between these two time points. The significance star(s) above the black line indicates the response from before to two hours after the bicycle challenge. B = before 45 min of bicycling, 0' = just after and 2 h = two hours after bicycling. w0 = before 12 weeks of exercise. w12 = after 12 weeks of exercise intervention.

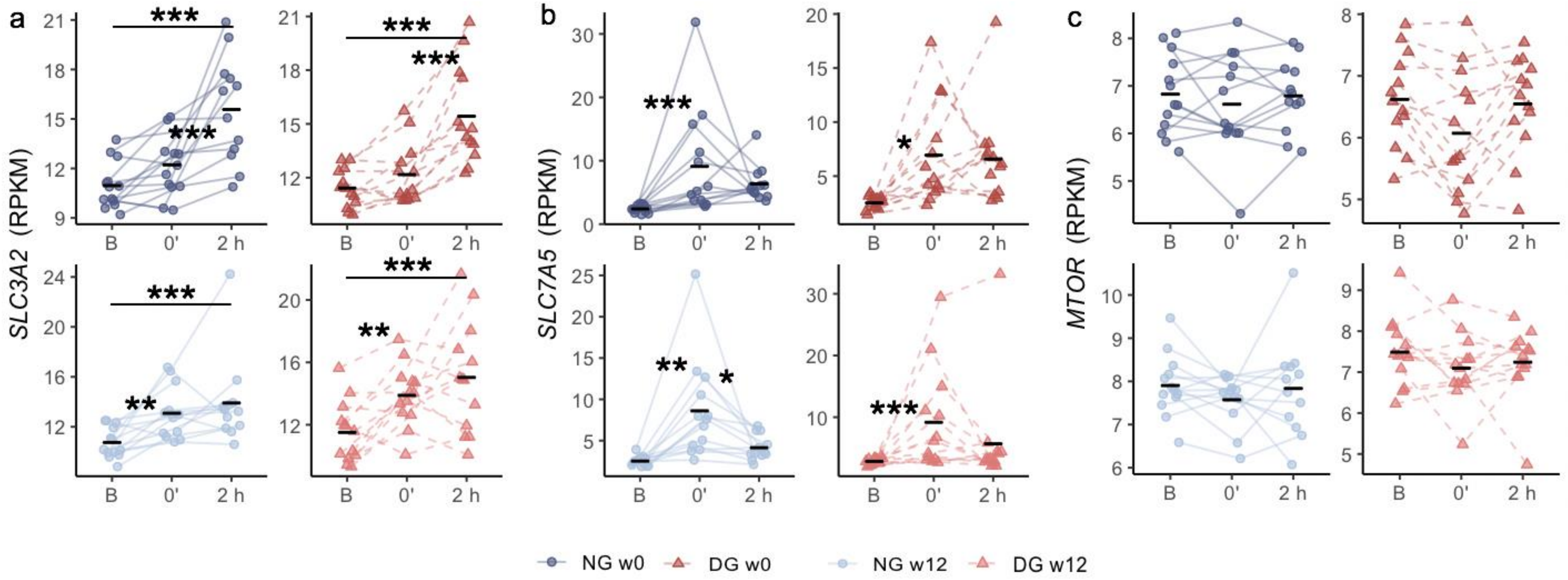

ESM Fig. 3. LAT1 and mTOR. (a-b) Transcript levels of the two components of the LAT1 amino acid transporter, and (c) mTOR in normoglycaemic (NG) and dysglycaemic (DG) men in response to a 45 min bicycling challenge before and after 12 weeks of exercise in skeletal muscle. *p<0.05, **p<0.01 and ***p<0.001. The significance star(s) between two time points indicates the response from the first to the second time point. The significance star(s) above the black line indicates the response from before to two hours after the bicycle challenge. B = before the acute 45 min bicycle challenge. 0' = just after the 45 min bicycle challenge. 2 h = two hours after the 45 min bicycle challenge. w0 = before and w12 = after 12 weeks of exercise. RPKM = reads per kilobase of transcript, per million mapped reads. The legend below the figure apply to all panels.

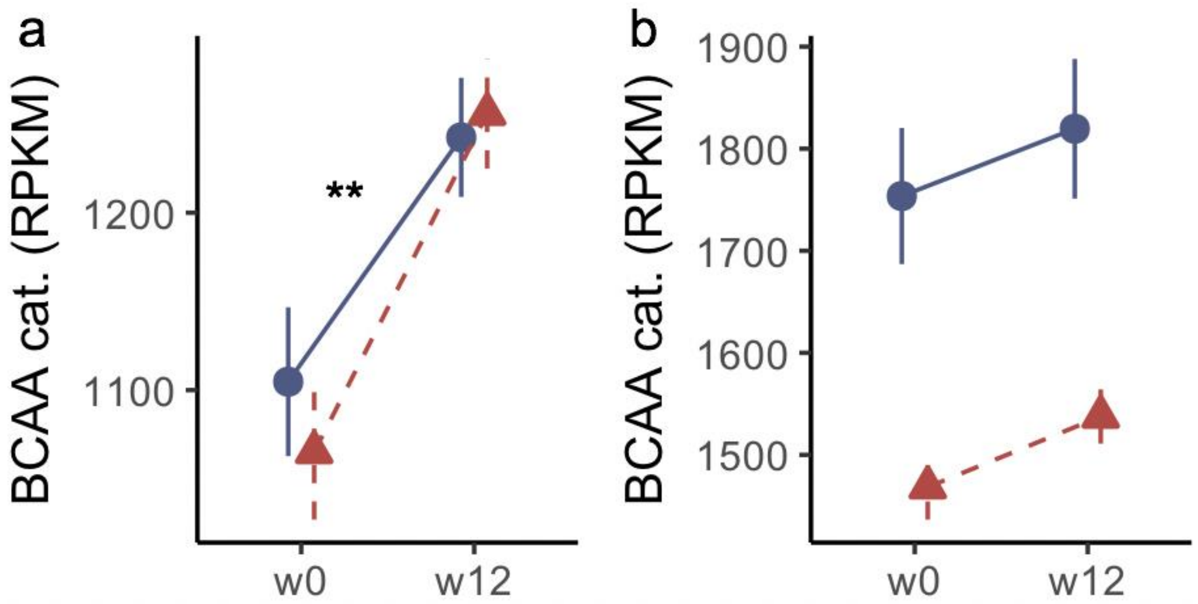

ESM Fig. 4. Group by time interactions. (a) Interaction effect between DG (red) and NG (blue) men for the change in median mRNA levels of all BCAA catabolic genes in response to 12 weeks of exercise in SkM, and (b) in ScWAT. **p<0.01 for the group by time interaction. w0 = before and w12 = after 12 weeks of exercise intervention. Cat. = catabolism. RPKM = reads per kilobase of transcript, per million mapped reads.

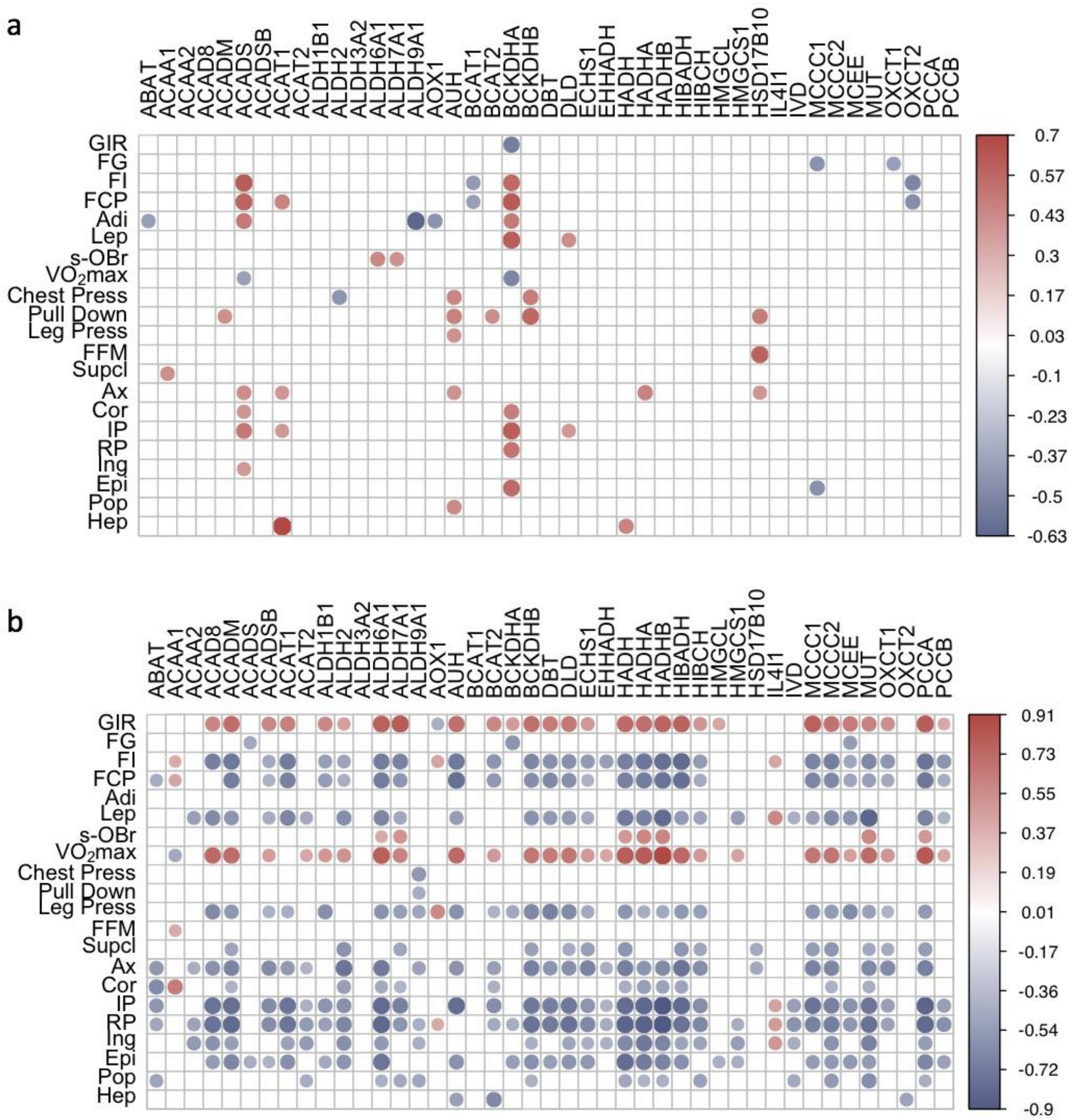

ESM Fig. 5. Correlations between mRNA levels of branched-chain amino acid (BCAA) catabolic genes in skeletal muscle (SkM) and subcutaneous white adipose tissue (ScWAT) and relevant phenotypes using data after 12 weeks of exercise. (a) Spearman's correlation in SkM, and (b) in ScWAT. The colour keys indicate Spearman's rho. Insignificant correlations are not shown. F = fasting. G = glucose. I = insulin. CP = C-peptide. Adi = high-molecular weight adiponectin. Lep = leptin. s-OBr = soluble leptin receptor. FFM = fat free mass. Supcl = supraclavicular fat. Ax = Axillary fat. Cor = cordial fat. IP = intraperitoneal fat. RP = retroperitoneal fat. Ing = inguinal fat. Epi = epidydimal fat. Pop = popliteal fat. Hep = hepatic fat. AU = arbitrary units. L = litres.

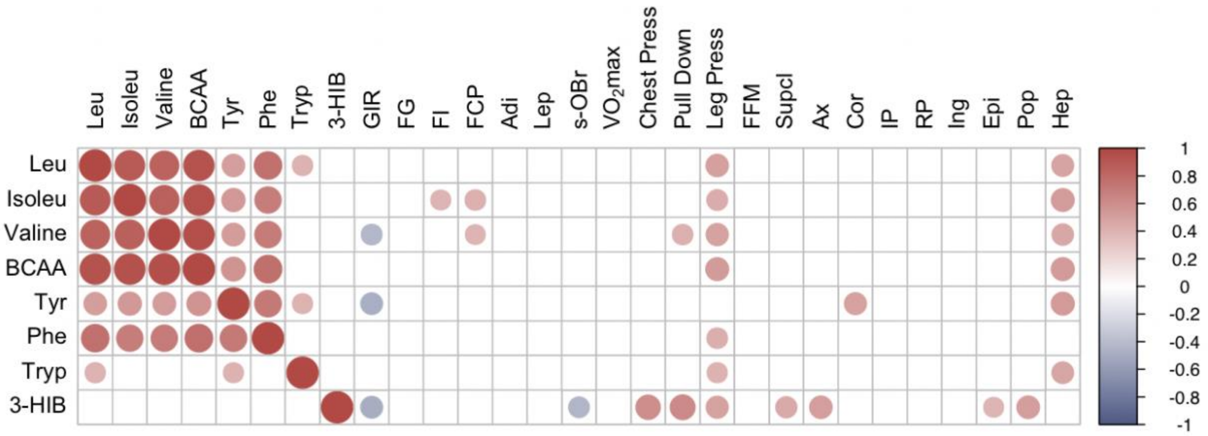

ESM Fig. 6. Plasma branched chain (BCAA) and aromatic amino acids after 12 weeks. A Spearman's correlation heatmap between plasma amino acids, 3-HIB, and relevant phenotypes after 12 weeks of exercise. The colour key to the right of the heatmap indicates positive (red) and negative (blue) correlations. Non-significant correlations are not shown. Trp = tryptophan. Phe = phenylalanine. Leu = leucine. Isoleu = isoleucine. GIR = glucose infusion rate. F = fasting. G = glucose. I = insulin. CP = C-peptide. Adi = high-molecular weight adiponectin. Lep = leptin. s-OBr = soluble leptin receptor. FFM = fat free mass. Supcl = supraclavicular fat. Ax = Axillary fat. Cor = cordial fat. IP = intraperitoneal fat. RP = retroperitoneal fat. Ing = inguinal fat. Epi = epidydimal fat. Pop = popliteal fat. Hep = hepatic fat. AU = arbitrary units. L = litres.

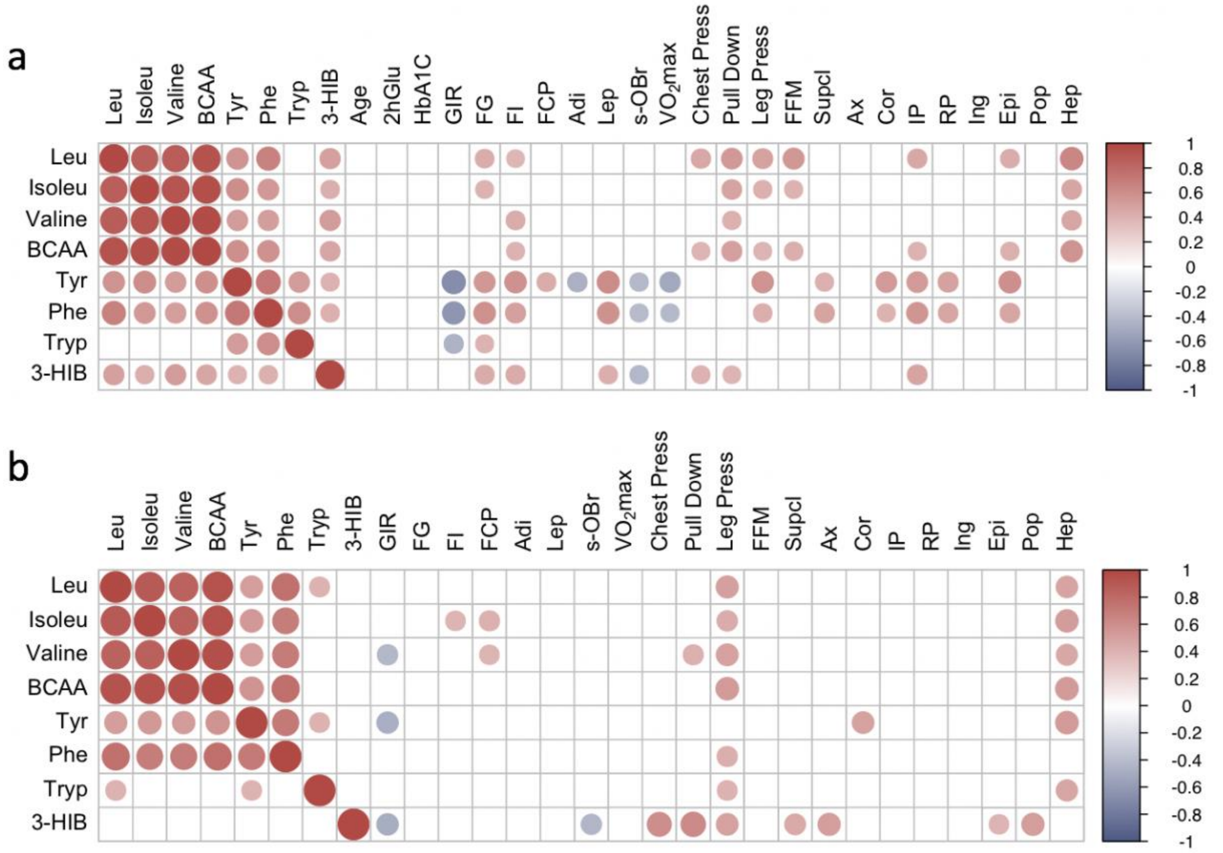

ESM Fig. 7. Plasma branched chain (BCAA) and aromatic amino acids correlations with phenotypes, corrected for fat free mass (FFM). (A) A Spearman's correlation heatmap between plasma amino acids, 3-HIB, and relevant phenotypes at baseline and (B) after 12 weeks of exercise. The colour key to the right of the heatmap indicates positive (red) and negative (blue correlations. Non-significant correlations are not shown. Trp = tryptophan. Phe = phenylalanine. Leu = leucine. Isoleu = isoleucine. 2hGlu = 2 h glucose after an oral glucose tolerance test. GIR = glucose infusion rate. F = fasting. G = glucose. I = insulin. CP = C-peptide. Adi = high-molecular weight adiponectin. Lep = leptin. s-OBr = soluble leptin receptor. Supcl = supraclavicular fat. Ax = Axillary fat. Cor = cordial fat. IP = intraperitoneal fat. RP = retroperitoneal fat. Ing = inguinal fat. Epi = epidydimal fat. Pop = popliteal fat. Hep = hepatic fat. AU = arbitrary units. L = litres.

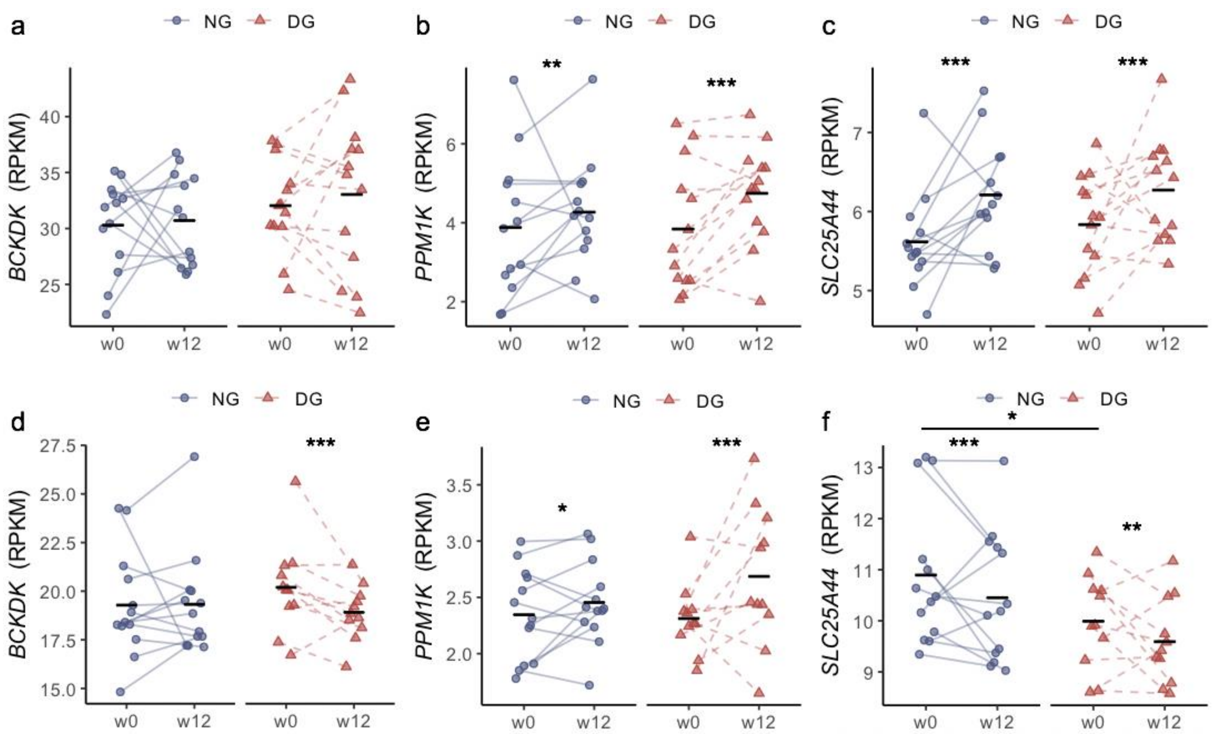

ESM Fig. 8. BCKDH kinase and phosphatase, and the SLC25A44 mitochondrial BCAA transporter mRNA. (a-c) Results in skeletal muscle, and (d-f) in subcutaneous white adipose tissue.*p<0.05, **p<0.01 and ***p<0.001. The significance star(s) placed between two timepoints indicates statistical significance of the response between these two time points. The significance star(s) above the black line indicates group difference at baseline.
